# An Integrated Approach to Protein Discovery and Detection From Complex Biofluids

**DOI:** 10.1016/j.mcpro.2023.100590

**Published:** 2023-06-09

**Authors:** Gordon T. Luu, Chang Ge, Yisha Tang, Kailiang Li, Stephanie M. Cologna, Andrew K. Godwin, Joanna E. Burdette, Judith Su, Laura M. Sanchez

**Affiliations:** 1 Department of Chemistry and Biochemistry, University of California Santa Cruz, Santa Cruz, California, USA; 2 Wyant College of Optical Sciences, University of Arizona, Tucson, Arizona, USA; 3 Department of Biomedical Engineering, University of Arizona, Tucson, Arizona, USA; 4 Department of Pharmaceutical Sciences, University of Illinois at Chicago, Chicago, Illinois, USA; 5 Department of Chemistry, University of Illinois at Chicago, Chicago, Illinois, USA; 6 Department of Pathology and Laboratory Medicine, University of Kansas Medical Center, Kansas City, Kansas, USA; 7 Kansas Institute for Precision Medicine, University of Kansas Medical Center, Kansas City, Kansas, USA; 8 The University of Kansas Cancer Center, Kansas City, Kansas, USA

**Keywords:** mass spectrometry, optical resonators, biomarkers, ovarian cancer, murine model

## Abstract

Ovarian cancer, a leading cause of cancer-related deaths among women, has been notoriously difficult to screen for and diagnose early, as early detection significantly improves survival. Researchers and clinicians seek routinely usable and noninvasive screening methods; however, available methods (*i.e.*, biomarker screening) lack desirable sensitivity/specificity. The most fatal form, high-grade serous ovarian cancer, often originate in the fallopian tube; therefore, sampling from the vaginal environment provides more proximal sources for tumor detection. To address these shortcomings and leverage proximal sampling, we developed an untargeted mass spectrometry microprotein profiling method and identified cystatin A, which was validated in an animal model. To overcome the limits of detection inherent to mass spectrometry, we demonstrated that cystatin A is present at 100 pM concentrations using a label-free microtoroid resonator and translated our workflow to patient-derived clinical samples, highlighting the potential utility of early stage detection where biomarker levels would be low.

## Highlights

• A workflow was to identify high-grade serous ovarian cancer biomarkers
• Mass spectrometry proteomics identified cystatin A
• A surface conjugated antibody for cystatin A facilitated pM microtoroid detection

## Graphical Abstract

**Figure:**
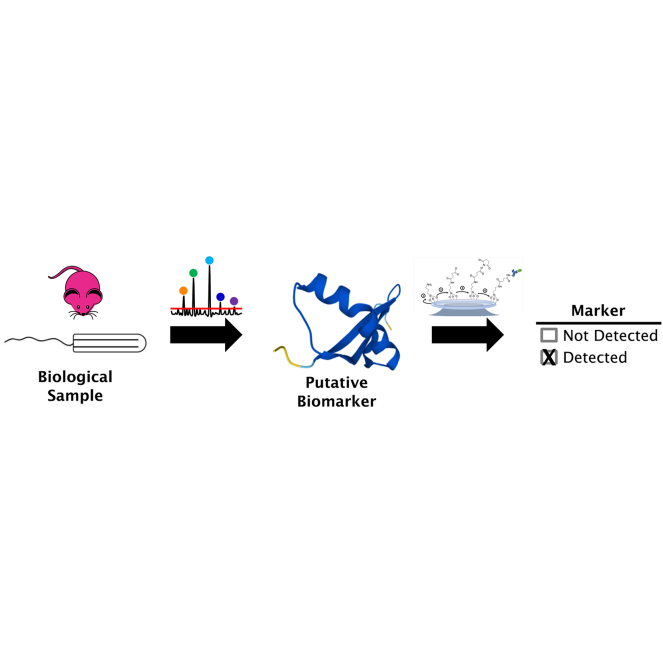

## In Brief

High-grade serous ovarian cancer can originate in the fallopian tube epithelium. Tumors colonize the ovary and then metastasize throughout the peritoneum; however, no routine screening exists for routine health exams. We leveraged vaginal lavages from a murine model as a complex biological fluid for protein biomarker discovery. We discovered and validated cystatin A as a putative biomarker. Detection was improved using a cystatin A antibody conjugated to a microtoroid resonator’s surface, facilitating detection from vaginal lavages and patient-derived tampons.

Ovarian cancer remains one of the most lethal gynecological cancers in women, with an estimated 19,880 new cases in the United States in 2022; it is second only to uterine cancer, which had 65,950 estimated new cases in 2022 (1). Because of a paucity of early detection strategies, the 5-year relative survival rate from 2011 to 2017 for ovarian cancer (49.1%) was nearly half that of uterine cancer (81.1%) (https://seer.cancer.gov/statfacts/html/ovary.html, https://seer.cancer.gov/statfacts/html/corp.html, https://seer.cancer.gov/statfacts/html/common.html). When detected early that the survival chance for ovarian cancer can increase to almost 90% (https://seer.cancer.gov/statfacts/html/ovary.html). Ovarian cancer seldom displays clinical symptoms prior to metastasis, leaving patients unaware of their condition until the disease progresses to stage III or IV. In addition, currently available screening methods do not detect the disease early (*i.e.*, transvaginal ultrasounds, CA-125) or lack the specificity and sensitivity to be used routinely (*i.e.*, human epididymis protein 4, OVA1, Overa) (2, 3, 4, 5, 6). Therefore, there is an urgent need for (1) reliable biomarkers accompanied by methodologies to detect them and (2) novel sampling methods to routinely screen for early stage gynecological cancers.

Brinton *et al.* (7) suggested that local tumor microenvironments may be a more appropriate alternative sampling source for ovarian cancer biomarkers as they would prove to be more useful in the detection of primary tumors, which cannot be done using metastatic biomarkers. To that end, Costas *et al.* (8) have previously suggested novel sampling methods in the context of endometrial cancer and outlined several criteria for effective sampling, including (1) high throughput, (2) able to detect early stages of disease, (3) minimally invasive, and (4) affordable. Vaginal sampling may provide a site where tumors that arise in the fallopian tube (*i.e.*, high-grade serous ovarian cancer, a prominent and particularly fatal subtype of ovarian cancer) or in the uterus (*i.e.*, endometrial cancer) may escape and concentrate (9, 10, 11).

**Figure 1:**
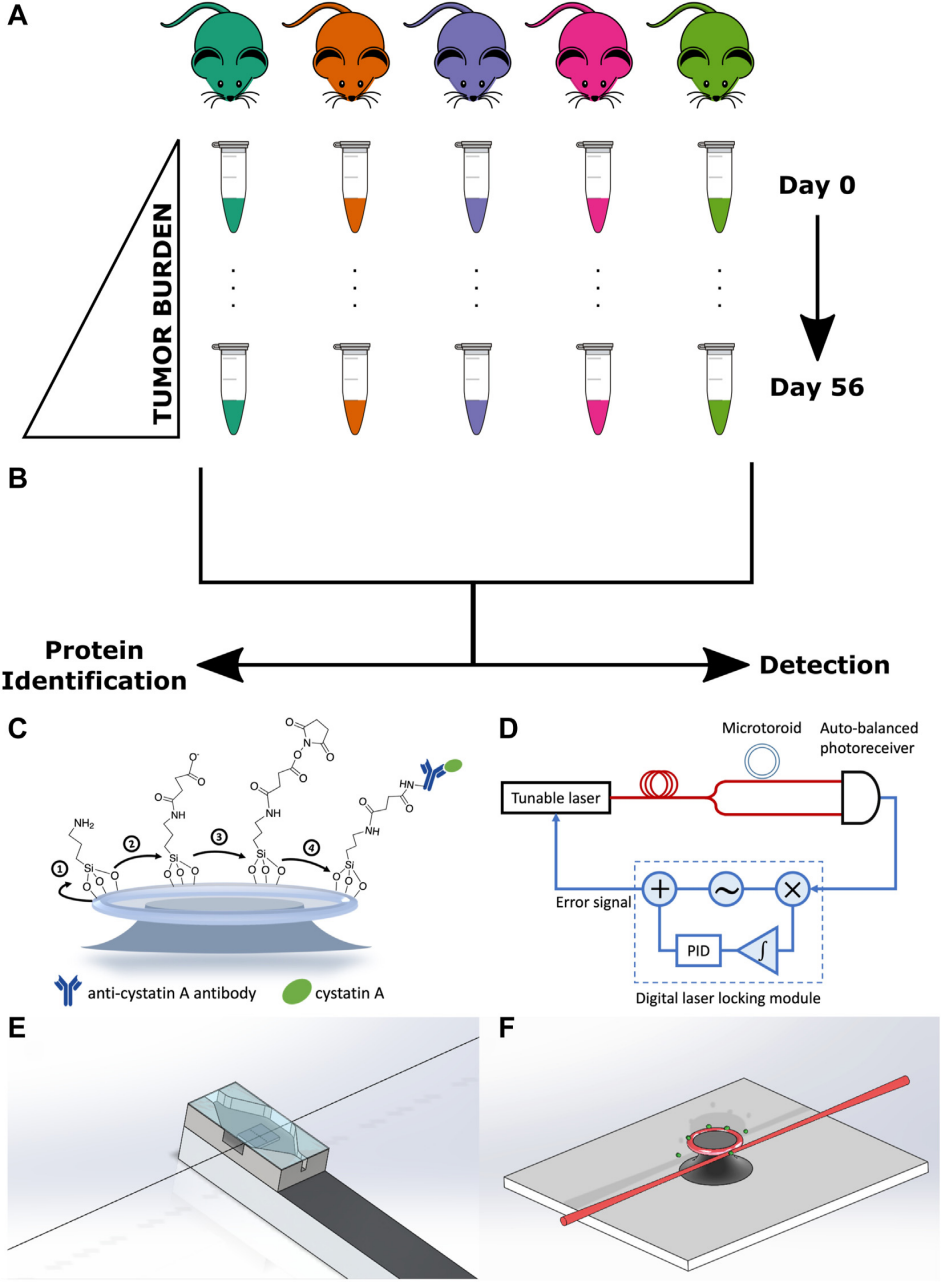
**Summary of the discovery workflow and microtoroid experimental setup**. *A*, previous study by Galey *et al.* collected murine vaginal lavages for xenograft OVCAR-8-RFP mice over 8 weeks and profiled each lavage using MALDI-TOF mass spectrometry (MS). *B*, the current study seeks to identify proteins detected from MALDI-TOF MS protein profiles and use more sensitive detection techniques. *C*–*F*, schematic of cystatin A biosensing experimental setup using FLOWER. *C*, preparation of the microtoroid for binding to the cystatin A–Ab complex. *D*, block diagram of the FLOWER system. A digital laser locking module enables adaptive tracking of the microtoroid’s resonance frequency, which changes as analytes bind to its surface. *E*, schematic representation of the microfluidic chamber designed to allow the passage of the optical fiber. *F*, an isometric view showing analyte molecules (*green*) binding to the surface of the toroid. The toroid is shown positioned next to a tapered optical fiber for evanescent coupling of light into the toroid.

We have previously used vaginal lavages to collect intact cells and extracellular proteins from the vaginal microenvironment of mice with OVCAR-8-red fluorescent protein (RFP) tumors, which models high-grade serous ovarian cancer (Fig. 1*A*) (9). Vaginal lavages collected over 8 weeks from a cohort of five mice were analyzed using MALDI-TOF mass spectrometry (MS) to profile the microprotein range, which consists of proteins <30 kDa. Routine monitoring of the menstrual cycle of mice and rats, which is approximately 4 days long and results in naturally occurring cell turnover event, is performed using vaginal lavages and is considered a safe procedure. Here, earlier time points (days 7–28 post tumor implantation) and later time points (days 35–56 post tumor implantation) represented early stage and late-stage ovarian cancer, respectively; day 0 time point occurred prior to tumor implantation and represented healthy mice. Following tumor progression, differentially expressed microproteins between early and later time points were identified. Importantly, our previous study analyzing a nonalcoholic steatohepatitis murine model indicated that routine sampling in a murine model with another inflammatory condition did not produce the same signatures as ovarian cancer, meaning the signals detected were not a byproduct of the vaginal lavage procedure (9). However, a limitation of MALDI-TOF MS protein profiling is that it only provides a putative intact mass with no other information on protein identity (2).

Herein, we have used LC–MS/MS based bottom–up proteomics to analyze the same murine lavages reported by Galey *et al.* to identify these putative biomarker features (Fig. 1*B*). In doing so, we have identified cystatin A and confirmed its presence in tumors and murine reproductive tissue, which provided insight into its spatial distribution *in vivo*. Knowing that MALDI-TOF MS provides a limited means for early detection for specific biomarkers over the multidimensional fingerprinting, we sought to integrate more sensitive and specific technologies for detection from the same lavage samples. Therefore, we applied a targeted approach to leverage frequency-locked optical whispering evanescent resonator (FLOWER) as a potential alternative early stage screening method (Fig. 1*B*) (12, 13, 14). FLOWER is based on microtoroid optical resonator technology and allows for label-free detection of potentially attomolar concentrations of an analyte, which is not possible with ELISA or MS (Fig. 1, *C*–*E*) (14, 15, 16). With FLOWER, light is evanescently coupled into the microtoroid using a tapered optical fiber. At the resonance frequency of the microtoroid, there will be a dip in the transmission of the light that passes through the optical fiber. This transmission dip is monitored as analyte molecules bind to antibodies anchored to the surface of the toroid. One advantage of using FLOWER for these experiments is its ultrasensitivity and compatibility with small (microliter) amounts of biofluids (*i.e.*, murine vaginal lavages) (15, 16, 17). In sum, we present a workflow for combining powerful untargeted technologies to discover microproteins for use with specific and sensitive label-free targeted screening. Furthermore, we have translated this workflow for use with patient-derived protein extracts sourced from tampons worn by women predebulking surgery.

## Experimental Procedures

### Chemicals

Aminopropyl trimethoxysilane (catalog no.: 440140), acetic acid (catalog no.: 695092), succinic anhydride (catalog no.: 239690), dimethyl sulfoxide (DMSO; catalog no.: 276855), anhydrous ethanol (catalog no.: 676829), and ethanolamine (catalog no.: E6133) were purchased from Sigma–Aldrich. Pierce premium grade *N*-hydroxysulfosuccinimide (sulfo-NHS; catalog no.: PG82071) and 1-ethyl-3-(3-dimethylaminopropyl) carbodiimide (EDC; catalog no.: 22980) were purchased from Thermo Fisher Scientific.

### *In vivo* Murine Xenograft Study

Full details for the *in vivo* murine xenograft study have been previously reported (9). All animals were humanely treated as approved by the Animal Care and Use Committee at the University of Illinois at Chicago using protocol #17-174. During the study, the collection of vaginal lavages (∼200 μl) for each mouse (numbered 901–905; N = 5) were collected into 1.5 ml microcentrifuge tubes at time points every 7 days starting on day 0 and ending on day 56 in a murine biological research laboratory with sterile PBS, which is not an irritant. It should be noted that while the PBS used was sterile, the vaginal environment is inherently not sterile and could not be sterilized. Furthermore, although 200 μl of lavage fluid was used, loss of fluid in the murine vagina and/or during the lavage procedure resulted in reduction of the total working volume collected, though this loss is minimal; this may result from movement of the mice as they are not anesthetized for this procedure. Care is taken to gently flush the murine vaginal canal while also ensuring that the procedure is relatively fast with minimal animal handling. In the present study, lavages from each mouse collected on day 0 acted as a control since this time point preceded tumor implantation.

### Storage of Murine Vaginal Lavages

Murine vaginal lavages for mice 901 and 902 were normalized and concentrated to 10,000 cells/μl, whereas mice 903, 904, and 905 were normalized and concentrated to 5000 cells/μl; the total concentrated volume of samples ranged from 4 to 20 μl, with one sample being 1 μl. All lavages were stored at −80 °C until used. Day 7, 21, 35, and 49 lavages were used for LC–MS/MS-based bottom–up proteomics, whereas day 14, 28, 42, and 56 lavages were shipped and used for detection of cystatin A *via* FLOWER. Upon receiving, the murine vaginal lavages were stored at −80 °C.

### Preparation of Murine Vaginal Lavages for Enzymatic Digestion

Protein concentration for samples was determined using a bicinchoninic acid (BCA) assay. Three biological replicates were pooled by time point. Pooled samples were diluted to a total volume of 500 μl, transferred to Amicon Ultra-0.5 30 kDa centrifugal filter devices (MilliporeSigma), and centrifuged at 14,000*g* for 10 min. Centrifugation was repeated with one 100 μl and two 50 μl PBS washes under the same conditions to ensure adequate sample collection. The retentate was collected by inverting the spin column filter and centrifuging at 1000*g* for 2 min. The retentate and filtrate concentrated *in vacuo* at 45 °C for 1 h.

### Enzymatic Digestion of Murine Vaginal Lavages Using S-Trap

A modified filter-aided sample preparation protocol using the S-Trap (ProtiFi) was used for enzymatic digestion of all pooled murine vaginal lavages. Previously collected filtrate from spin column filter for pooled lavages for odd time points were resuspended in 25 μl 10% sodium dodecyl sulfate solution with a protease inhibitor cocktail (Roche). Reduction of disulfides was performed by adding DTT to the lavages to a final concentration of 20 mM and heating at 95 °C for 10 min. After cooling to room temperature (RT), alkylation of cysteines was performed by adding iodoacetamide to a final concentration of 40 mM and incubating in the dark for 30 min. Lavages were then centrifuged at 13,000*g* for 8 min to remove any undissolved matter. About 12% aqueous phosphoric acid was added at 1:10 and mixed for a final concentration of 1.2% phosphoric acid. About 165 μl of triethylammonium bicarbonate in 90:10 methanol:MilliQ water (S-Trap buffer) was added to lysed lavages and mixed. The mixture was then added to the S-Trap. The S-Trap was centrifuged at 2000*g* for 30 s to elute the S-Trap buffer and retain proteins in the S-Trap column. About 150 μl of S-Trap buffer was added to the S-Trap and centrifuged at 2000*g* for 30 s; this process was repeated five times with the S-Trap column being rotated 180° between repeated centrifugations. rLys-C (Promega) was added at 1:50 weight:weight to the S-Trap column, which was placed in a clean microcentrifuge tube. Digestion was performed by incubating at 37 °C overnight. After digestion, the following solutions were added to the S-Trap in sequential order to elute peptides: (1) 40 μl 50 mM triethylammonium bicarbonate in MilliQ water followed by centrifugation at 4000*g* for 30 s, (2) 40 μl 0.2% formic acid in MilliQ water followed by centrifugation at 4000*g* for 30 s, and (3) 35 μl of 60:40 acetonitrile (ACN):MilliQ water followed by centrifugation at 4000*g* for 1 min. Eluted peptide solutions were dried *in vacuo* at 60 °C for 1 h.

### Enzymatic Digestion of Murine Vaginal Lavages Using in-Solution Digestion

In-solution enzymatic digestion was also performed on a small aliquot of pooled murine vaginal lavages for days 35 and 49. Lavages were reduced and alkylated as outlined in the aforementioned S-Trap digestion protocol. Following reduction and alkylation, rLys-C was added at 1:50 weight:weight to the lavage, which were then incubated at 37 °C overnight and dried *in vacuo* at 60 °C for 1 h.

### Sample Desalting and Preparation for LC–MS/MS

Following enzymatic digestion, samples were resuspended in 100 μl 0.1% formic acid in MilliQ water. Samples were then desalted using C18 Resin ZipTip pipette tips (MilliporeSigma) per the manufacture’s protocol, and desalted samples were dried *in vacuo* at 45 °C for 30 min. Samples were resuspended in 0.1% formic acid to a concentration of 0.5 μg/μl and centrifuged at 13,000*g* for 10 min to remove particulates before being transferred to plastic LC–MS/MS vials. A 0.1% formic acid blank was also prepared.

### Data Acquisition *via* LC–MS/MS

Analysis was performed on a Q Exactive mass spectrometer (Thermo Fisher Scientific) coupled to an Agilent 1260 Infinity nanoLC system (Agilent Technologies). Samples were loaded onto an Acclaim Pepmap 100 C18 trap column (75 μm × 2 cm nanoViper, 3 μm, 100 Å) (Thermo Fisher Scientific) at 2 μl/min. After 10 min of washing with water with 0.1% formic acid (solvent A), separation was performed using a 60 min gradient at a flow rate of 0.25 μl/min on a Zorbax 300SB-C18 column (0.075 × 150 mm, 3.5 μm, 300 Å). The gradient was as follows: 60 min from 5 to 30% ACN with 0.1% formic acid (solvent B) and then 20 min from 30 to 60% B. The system was then maintained at 90% B for 10 min followed by a 15 min re-equilibration segment at 5% B. Data-dependent acquisition was used during the collection of mass spectra with a capillary temperature of 250 °C and spray voltage of 1.5 kV. Full MS scans were collected at a mass resolution of 70,000 with a scan range of *m/z* 375 to 2000. Automatic gain control target was set at 1e6 for a maximum injection time of 100 ms. The top ten most intense peaks were selected for MS/MS analysis, with an isolation width of 1.5 *m/z*. MS/MS spectra were acquired at a resolution of 17,500 with an automatic gain control target of 1e5 and maximum injection time of 50 ms. The first fixed mass was set at 100 *m/z*. Parent ions were fragmented at a normalized collision energy of 27. Dynamic exclusion was set for 20 s. Two technical replicates (n = 2) were collected for each pooled sample.

### Annotation of Microproteins Using MaxQuant

Label protein annotation was performed in MaxQuant (version 1.5.4.0) against proteomes for *Homo sapiens* (81,837 sequences), *Mus musculus* (55,286 sequences), and *Rattus norvegicus* (47,945 sequences) obtained from UniProt (release-2022-05). Default MaxQuant parameters were used unless otherwise specified, and Lys-C was specified as the digestion enzyme, allowing up to two missed cleavages. First and main peptide search tolerances were set to 20 ppm and 4.5 ppm, respectively (Orbitrap defaults). Label-free quantification was disabled because of insufficient replicates as a result of sample pooling. Carbamidomethylation was specified as a fixed modification, whereas oxidation and protein N-terminal acetylation were set as variable modifications, and a maximum of five modifications per peptide were allowed. The minimum peptide length allowed was set to seven, and the maximum peptide mass was 30 kDa. The minimum and maximum peptide lengths for unspecific search were set to 8 and 25, respectively. The peptide-spectrum match, protein, and site false discovery rates were all set to 1% as calculated by the target-decoy approach. Because of the low number of annotations, no thresholding was performed on MaxQuant scores prior to manual retrospective analysis of MALDI protein profiles.

### Retrospective Analysis of Murine Vaginal Lavage Protein Profiles Acquired *via* MALDI-TOF MS

Following annotation in MaxQuant, results were filtered to remove any annotations with a mass greater than 30 kDa, less than two unique peptides, and any contaminants, which resulted in a total of 13 proteins (Table 1). Posterior error probability values were calculated and appended to Table 1 using an R script found at https://github.com/pstew/maxquant_pepcalc.

**Table 1:** Curated list of proteins annotated from LC–MS/MS data processed *via* MaxQuant

Protein	Gene	Molecular weight (kDa)	Sequence coverage (%)	Unique sequence coverage (%)	Sequence length	PEP score	PEP
Caspase-14	CASP14	27.679	14.9	14.9	242	22.3	4.75e-11
Heat shock protein beta 1	HSPB1	22.782	19.0	19.0	205	11.1	7.93e-04
Histone H1.2/H1.3/H1.4	H1-2/H1-3/H1-4	21.316	16.0	16.0	212	18.2	6.50e-07
Calmodulin-like protein 5	CALML5	15.892	47.3	47.3	146	79.5	2.87e-60
**Fatty acid–binding protein 5**	**FABP5**	**15.164**	24.4	24.4	135	24.8	**1.44e-13**
**Protein S100-A9**	**S100A9**	**13.242**	24.6	24.6	114	32.8	**1.49e-25**
**Histone H2B1**	**H2B1**	**13.990**	16.0	16.0	125	42.8	**1.77e-13**
Protein S100-A7	S100A7	11.471	28.7	28.7	101	15.7	1.90e-08
**Histone H4**	**H4c1**	**11.367**	26.2	26.2	103	12.0	**9.65e-05**
Dermicidin	DCD	11.284	16.4	16.4	110	13.7	2.05e-06
**Cystatin-A**	**CSTA**	**11.006**	39.8	39.8	98	36.7	**1.99e-21**
**Protein S100-A8**	**S100A8**	**10.834**	45.2	45.2	93	42.7	**2.10e-23**
Ubiquitin-60S ribosomal protein L40		7.132	49.2	49.2	63	109.3	4.52e-102

Proteins with <2 unique peptides and >30 kDa were filtered out. Six proteins found in MALDI-TOF MS protein profiles are shaded in *bold*. *Q* values for all annotations (not shown) were found to be 0. MS/MS spectra for peptides used by MaxQuant for annotation are found in supplemental Figs. S9–S21. The PEP score is defined in MaxQuant as derived from peptide posterior error probabilities (PEPs), which have been described by Käll *et al.* (96). The PEP score calculated in MaxQuant is inversely related to the protein group PEPs and should be treated as a thresholded value. More detailed identification data can be found in supplementary File 2. Furthermore, a brief discussion of other microproteins of interest in the context of ovarian cancer can be found in the supplemental data.

Protein profiles for the vaginal lavages previously collected by Galey *et al.* were also manually inspected for each of the 13 proteins in R (9). A total of 1056 profiles were loaded using the MALDIquant, version 1.21 and MALDIquantForeign, version 0.13 R packages for data preprocessing (five mice, weekly lavages collected from day 0 to day 56, 24 technical replicates per lavage; day 56 lavage for mouse 904 was absent). Parameters for the following preprocessing steps were left at their default values unless otherwise specified. All spectra were trimmed to a range of 4000 to 20,000 Da. Intensity transformation was performed using the square root method. Baseline smoothing was performed using the SavitzkyGolay method. Baseline removal was performed using the TopHat method. Intensity normalization was performed using the total ion current method. Peak detection was performed using the minimum absolute deviation method of noise estimation and a signal-to-noise ratio of 3:1. Peak alignment was performed with a signal-to-noise ratio of 3:1 and a tolerance of 0.2 Da; peaks were excluded if they were found to occur in less than 75% of the dataset. Preprocessing yielded a feature matrix containing 1939 features.

**Figure 2:**
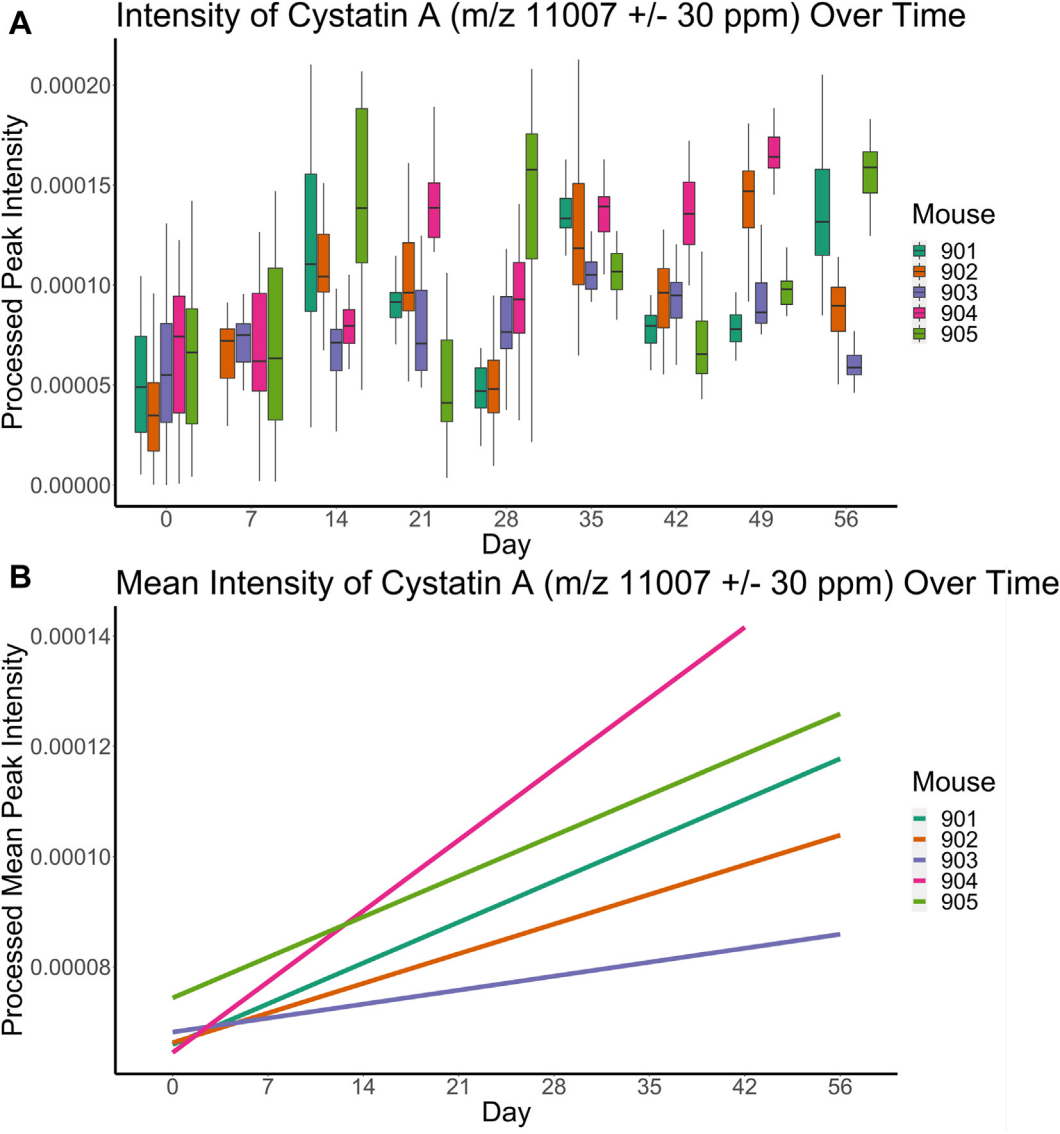
**Feature intensity as measured by MALDI-TOF mass spectrometry (MS) of *m/z* 11,007 (cystatin A) in protein profiles in five biological replicate xenograft murine models transfected with OVCAR-8-RFP tumors.***A*, box plot showing the intensity of cystatin A at each corresponding time point. A comparison between day 0 and day 56 samples using the Welch’s two-sample *t* test provided mean intensities of 0.000057 for day 0 and 0.00011 for day 56 (*p* < 2.2e-16; 95% confidence interval = [−0.000063, −0.000041]). *B*, linear regression trend lines for the mean intensities of cystatin A at each time point showing upregulation. The same plot can be found in supplemental Fig. S6 with the confidence intervals plotted.

Six proteins annotated by MaxQuant could be found in this feature list as highlighted in Table 1. Of these six features, intensity over time for cystatin A and protein S100-A8 was plotted in Figure 2*A* and supplemental Fig. S1*A*. Outlier detection using the interquartile range (IQR) method was attempted prior to plotting. Outlier removal performed on cystatin A removed 45 of the original 1056 data points (4.3% of total data points), whereas 80 of the original 1056 data points (7.6% of total data points) were removed for protein S100-A8. The regression of the mean intensity by day and mouse was also plotted using the ggplot2::geom_smooth function calculated with the “linear” method (Fig. 2*B* and supplemental Fig. S1*B*).

### Validation of Cystatin A From Murine Tumors and Tissue Using Immunohistochemistry

**Figure 3:**
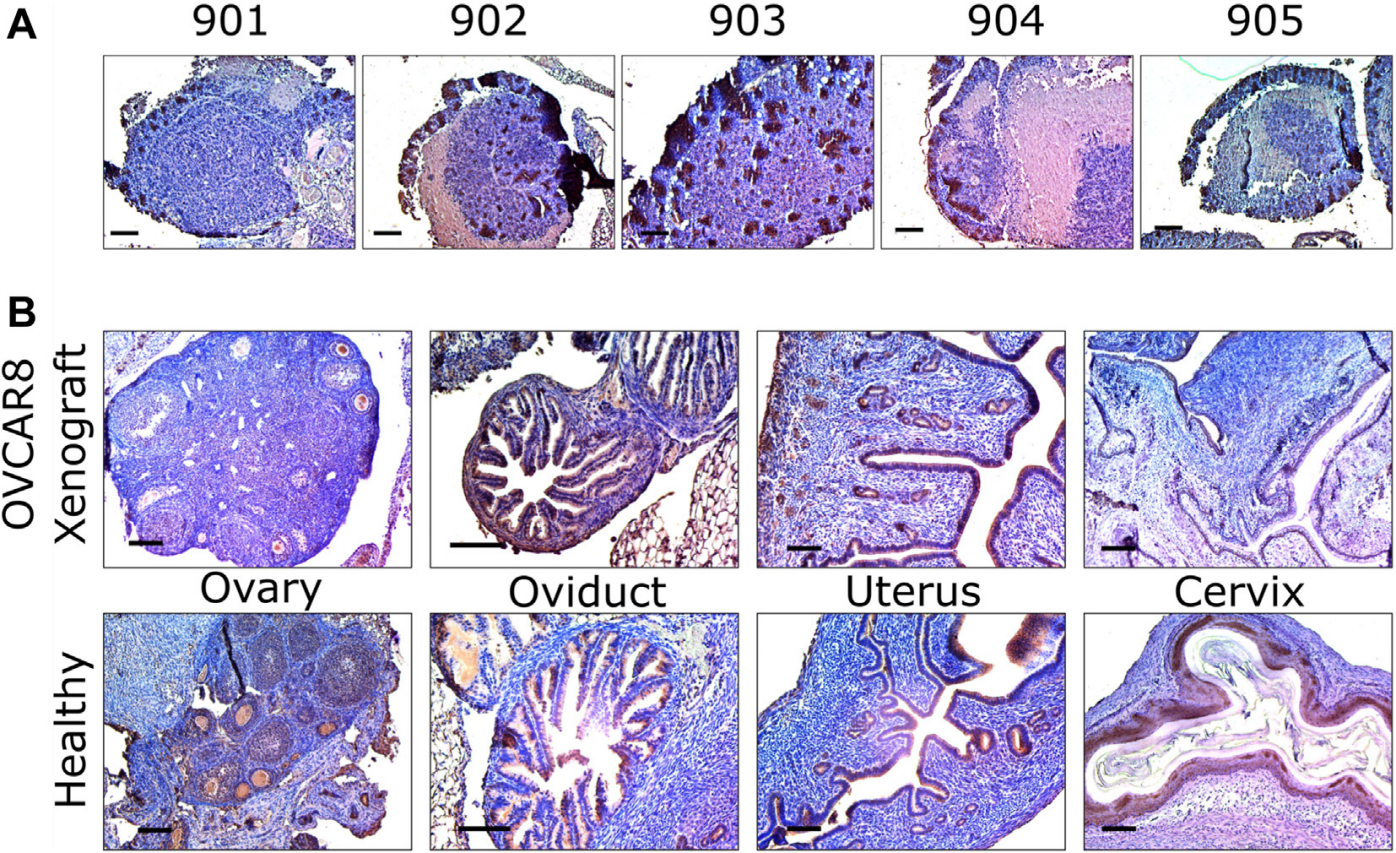
**Immunohistochemistry (IHC) staining.***A*, tumors from mouse 901 to 905. *B*, healthy and OVCAR8 xenograft murine reproductive tissue (ovary, oviduct, uterus, and cervix).

Tumor and malignant reproductive tissues were harvested from each mouse *via* necropsy performed on day 56 following collection of the final vaginal lavage with the exception of mouse 904, which expired prior to the end of the study (9); in the case of reproductive tissue, healthy tissues from other mice were also collected as a control. Slides of tumors and reproductive tissues were subjected to heat-induced antigen retrieval after deparaffinization using sodium citrate at 100 °C for 30 min and inactivation of endogenous peroxidase activity using 0.3% H_2_O_2_/methanol for 15 min. After rinsing with PBS with Tween-20 (PBST), the slides were blocked with 5% horse serum (Vectastain ABC kit; Vector Laboratories, Inc) diluted in 1% bovine serum albumin/PBST at RT for 60 min. The tissue sections were incubated with cystatin A primary rabbit polyclonal antibody (1:100 dilution, ThermoFisher; catalog no.: PA5-75206) corresponding to amino acids 51 to 100 of human cystatin A overnight at 4 °C. The next day, slides were rinsed with PBST prior to incubation with a biotinylated secondary antibody (Vectastain ABC kit; Vector Laboratories, Inc) at 1:200 dilution in PBST for 60 min at RT. Slides were then rinsed and incubated in ABC solution (PBS:A: B = 50:1:1) (Vectastain ABC kit) for 30 min at RT. For visualization of the immunoreactivity, all slides were subjected to chromogen 3′3-diaminobenzidine (Vector Laboratories, Inc) for 1 min and rinsed in running tap water for 10 min. Then slides were counterstained with hematoxylin and imaged using Nikon E600 Eclipse microscope with CMOS C-Mount microscope camera. The resulting images are found in Fig. 3.

### Preparation of Cystatin A Antibody Functionalized Microtoroid

To specifically detect cystatin A in mouse samples, recombinant rabbit monoclonal cystatin A antibodies (Thermo Fisher Scientific; catalog no.: MA5-29200) from rabbits immunized with human cystatin A were immobilized on the silica surface of the microtoroid through EDC/sulfo-NHS covalent coupling. Microtoroid optical resonators were fabricated as described previously using a combination of photolithography and etching steps (18). The final structure was formed by melting the silica disk edge with a CO_2_ laser. Microtoroids were treated with 1% v/v aminopropyl trimethoxysilane in 1 mM acetic acid for 10 min and incubated overnight with a solution of 100 mg/ml succinic anhydride in DMSO (Fig. 1C). After succinylation, the chip was rinsed with DMSO and ethanol before it was dried in a stream of nitrogen. EDC/sulfo-NHS 100 mM/100 mM in 0.1 M Mes buffer, pH 5, was prepared freshly and immediately applied on the chip. After 10 min of EDC/NHS conjugation, the chip was washed with 10 mM PBS, pH 7.4, and then incubated with cystatin A antibodies (50 μg/ml in PBS) for 1 h. The surface was subsequently quenched with 100 mM ethanolamine for 5 min to block residual amine-reactive groups. The antibody-coated chip was incubated in PBS buffer for further sensing experiments.

### Detection of Cystatin A From Murine Vaginal Lavages Using FLOWER

Immediately prior to running FLOWER experiments, samples were thawed and aliquoted; 4 to 5 μl was removed from each sample and diluted a 1000-fold for usage. The 1 μl sample was diluted 4000-fold. Remaining aliquots were flash-frozen in a cooling bath of dry ice/isopropyl acetone (BTC; catalog no.: 211315) and stored at −20 °C. FLOWER (Fig. 1, *C*–*F*) was used to investigate progressing cystatin A levels in murine vaginal lavage samples (14, 15, 16). The antibody-immobilized toroid chip is mounted on an open microfluidic chamber, which is designed to allow the passage of the optical fiber. Diluted murine samples are continuously perfused through the chamber at a steady flow rate of 100 μl/min. Here, the cystatin A antigen–antibody binding events are monitored in real time by adaptively tracking the resonance frequency of the microtoroid to obtain analyte binding curves. The monitoring of the shift in resonance wavelength of the microtoroid is recorded while steadily perfusing samples into the chamber. Each diluted murine sample is flowed for 5 min followed by 1 min of regeneration buffer (glycine–HCl, pH 3.0) to dissociate cystatin A from the antibodies and 5 min of sensing buffer (10 mM PBS, pH 7.4) rinsing.

**Figure 4:**
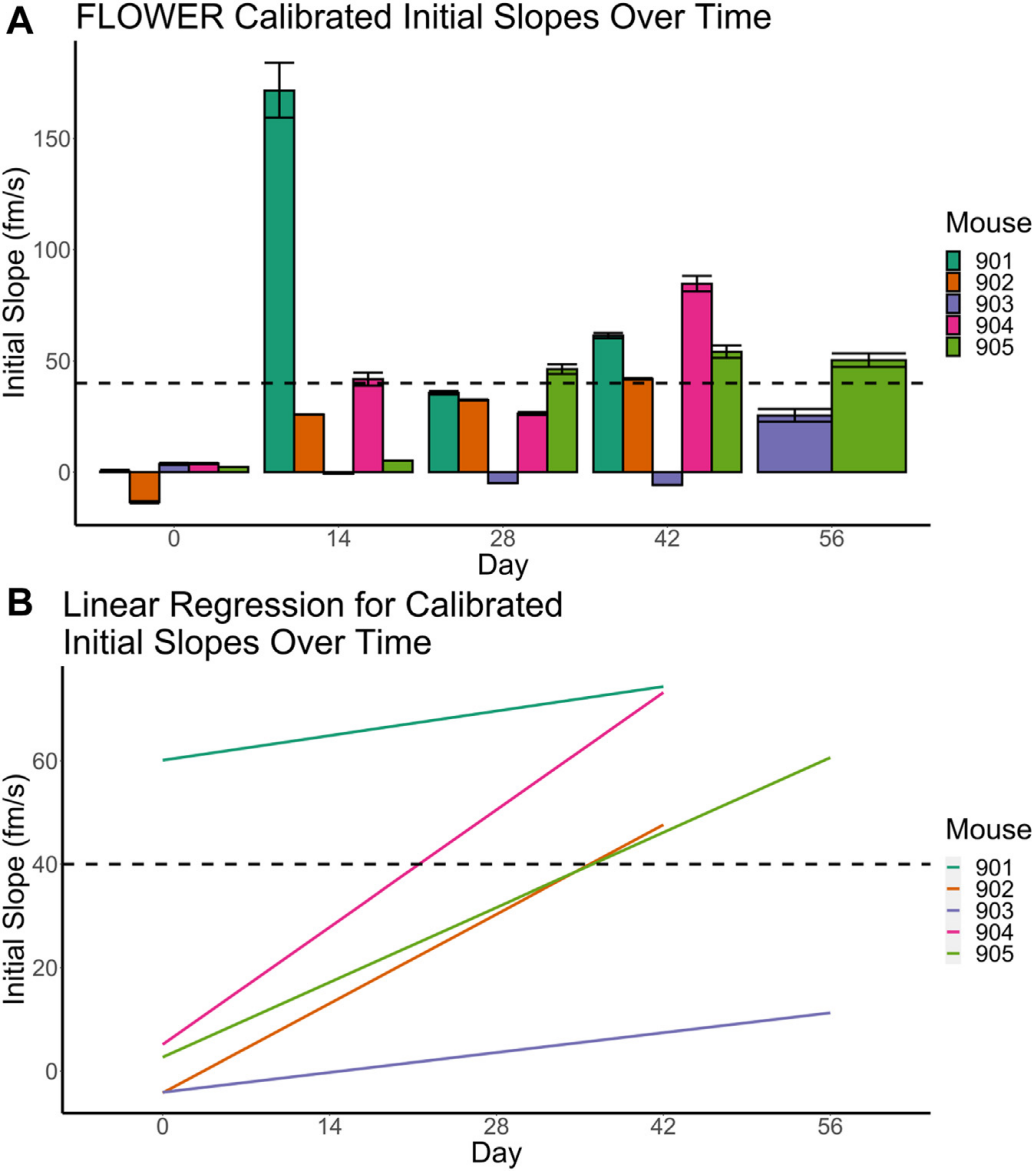
**Label-free detection of cystatin A using FLOWER.** The combined plot for all five mice samples with 100 pM calibrations. *A*, the line plot of the initial slope values as a function of days since tumor implantation. The *dashed line* indicates the value obtained from the normalized standard concentration of 100 pM. *B*, linearly fitted initial slope values *versus* time. The same plot can be found in supplemental Fig. S8 with the confidence intervals plotted.

The recordings that last for more than 1 h were segmented to extract the binding curves for murine samples from different time points; as a result, the use of single measurements means that the SEM is not available. A curve is fit to the lines using either Equations 1 or 2, and the maximum wavelength shift (λ_max_) plateau or highest point in the curve along with the observed rate constant (*k*_obs_) is used to determine the initial slope, which serves as the value for the binding rate to the functionalized microtoroid antibody (Fig. 4). Extracted curves are further calibrated by subtracting the nearby buffer background. A representative curve of cystatin A binding to anticystatin A in PBS is shown in supplemental Fig. S4.

Values recorded *via* FLOWER are inherently variable, with variability being based on (1) the size, geometry, and characteristics of different microtoroids, (2) the flow conditions of the sample, and (3) the immobilization efficiency. Therefore, all initial slope values are calibrated to a 100 pM standard of cystatin A to normalize the initial slopes and account for these variations.

### Microprotein Extraction From Deidentified Patient Tampons

Deidentified clinical samples were provided by the University of Kansas Medical Center’s Biospecimen Repository Core Facility (BRCF) along with the histologic diagnoses. Tampons were worn 1 day prior to tumor mass resection surgery by women enrolled under the KU repository’s Institutional Review Board–approved protocol (principal investigator: Godwin, HSC #5929) and following US Common Rule before collection. All tampon samples were stored at −80 °C until extraction.

The top one-fourth, or “head,” of the tampon was sectioned before being cut in half, resulting in a tampon section equivalent to one-eighth of the full tampon. Each tampon section was placed in a syringe and saturated with water for 10 min before being mechanically compressed to yield a crude protein extract; this process was repeated once. The crude extracts were transferred to Amicon Ultra-0.5 30 kDa centrifugal filter devices (MilliporeSigma) and centrifuged at 14,000*g* for 10 min. The retentate was also collected by inverting the Amicon spin column filter and centrifuging at 1000*g* for 2 min. The retentate and filtrate were collected into clean 2 ml microcentrifuge tubes and concentrated *via* lyophilization.

### Acquisition of Protein Profiles From Patient Tampons *via* MALDI-TOF MS

Sample preparation was performed using a modified procedure outlined by Petukhova *et al.* and Galey *et al.* Crude protein extracts were resuspended in MilliQ H_2_O at a concentration of 10 mg/ml and mixed in equal volume with 20 mg/ml sinapic acid in 70:30 ACN:MilliQ H_2_O with 0.1% trifluoroacetic acid and chilled on ice for 10 min to facilitate cocrystallization. Twenty-four 1.5 μl spots per extract were spotted onto 384-spot ground steel MALDI plates (Bruker Daltonics) and allowed to air dry. Two spectra were acquired per spot, yielding a total of 48 technical replicates. Protein Standard I (Bruker Daltonics) was used as an external calibrant. An Autoflex LRF MALDI-TOF mass spectrometer (Bruker Daltonics) was used to acquire mass spectra in positive linear mode with a mass range of 4 to 20 kDa using a laser power of 80%, detector gain of 18.1×, and laser width of 3 (medium) in flexControl v3.4 (Bruker Daltonics) in AutoXecute mode. About 4000 laser shots were accumulated in 50 shot increments for each sample with random walk enabled across the entire spot.

### Analysis of Patient Tampon Protein Profiles Acquired *via* MALDI-TOF MS

**Figure 5:**
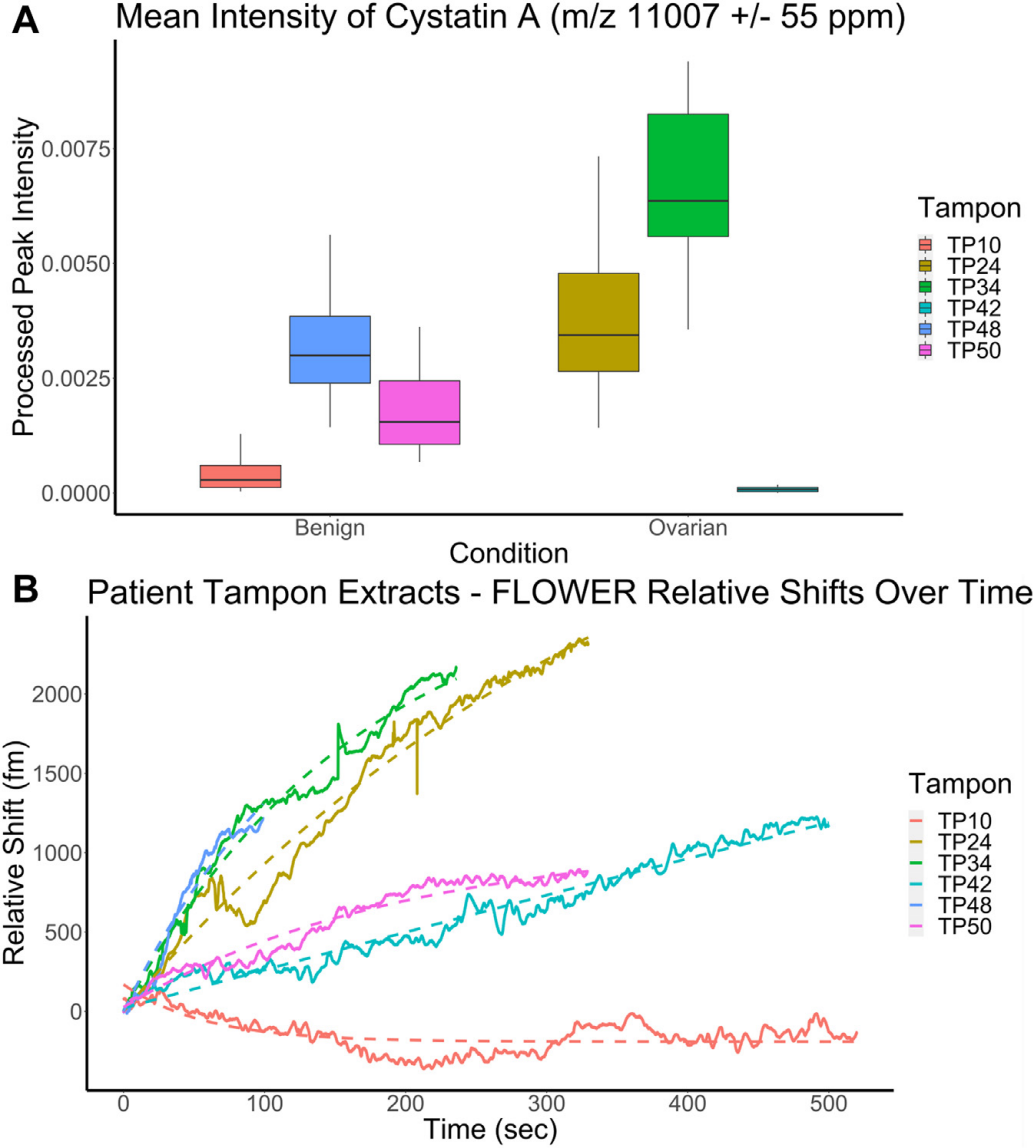
**Cystatin A detection from patient derived tampons.***A*, feature intensity as measured by MALDI-TOF mass spectrometry (MS) of *m/z* 11,007 (cystatin A) in protein profiles from six patient-derived tampon extracts following preprocessing and outlier removal. A comparison between benign and ovarian cancer extracts using Welch’s two-sample *t* test provided mean intensities of 0.0018 for benign cancer and 0.0034 for ovarian cancer (*p* < 1.07e-07; 95% confidence interval = [−0.0021, −0.0010]). *B*, label-free detection of cystatin A from patient-derived tampon extracts using a microtoroid resonator. We observe that the amount of cystatin A as measured by MALDI-TOF MS protein profiling agrees with measurements *via* FLOWER (frequency-locked optical whispering evanescent resonator).

To detect cystatin A, protein profiles for the patient-derived tampons were loaded and processed as outlined previously except for peak alignment. Peak alignment was performed with a signal-to-noise ratio of 3:1 and a tolerance of 0.2 Da; peaks were excluded if they were found to occur in less than 25% of the dataset. Preprocessing yielded a feature matrix containing 1754 features. The mean intensity of cystatin A was plotted alongside the estimated concentration for each tampon following outlier detection *via* the IQR method and removal (Fig. 5*A*).

### Detection of Cystatin A From Patient-Derived Tampon Extracts Using FLOWER

Patient-derived tampon extracts were handled as described previously prior to FLOWER experiments. Working solutions for patient-derived tampon extracts contained 1 μg/ml of each extract resolubilized in 10 mM PBS, pH 7.4. FLOWER was used as described previously; however, each diluted sample was flowed over the functionalized microtoroid chip for 5 to 10 min.

### Experimental Design and Statistical Rationale

Five biological replicates were collected for murine vaginal lavage experiments at all time points to ensure adequate statistical power. Furthermore, 24 technical replicates for each time point were previously acquired MALDI protein profiles by Galey *et al.* with the exception of day 56 for mouse 904 because of lack of adequate sample during collection. Analysis of MALDI protein profiles collected for murine vaginal lavages was subject to a standard preprocessing workflow in MALDIquant, version 1.21 per its documentation followed by outlier removal *via* the IQR method when calculating cystatin A abundance to account for run to run variation.

Because of the relatively low protein concentrations found in murine vaginal lavages, the samples were pooled by time point for odd numbered weeks (days 7, 21, 35, 49) prior to LC–MS/MS analysis to ensure that protein concentrations were above the limit of detection as determined by BCA. Even numbered weeks were saved for targeted detection of cystatin A *via* FLOWER. Digestion was performed using Lys-C, which cleaves proteins at the C-terminal region of lysine residues, as our protease of choice to ensure excess peptidic digestion did not occur.

Three patient-derived tampons for each diagnosis, benign masses or with a primary cancer of ovarian cancer, were analyzed as a proof of principle. Furthermore, 48 technical replicates for each sample were collected to account for the relatively low sample size. Analysis of MALDI protein profiles was the same as described previously.

## Results

### Annotation of Differentially Expressed Microproteins in Murine Vaginal Lavages *via* LC–MS/MS-Based Bottom–Up Proteomics

To annotate the microproteins from murine vaginal lavages previously collected by Galey *et al.* (9), LC–MS/MS-based bottom–up proteomics was used with the goal of identifying the putative proteins detected *via* MALDI-TOF MS screening. It should be noted that a typical murine vaginal lavage has a volume less than 200 μl with variable amounts of biological material (*i.e.*, cells, proteins) across biological replicates. Because of the limited sample volumes, lavages from mouse 903, 904, and 905 were pooled by alternating time points (days 7, 21, 35, and 49) for proteomics experiments, yielding pooled samples at day 7, 21, 35, and 49 postxenograft. Limited sample digestion was performed with a modified filter-aided sample preparation protocol with Lys-C as the digestion enzyme. In solution, enzymatic digestion for pooled day 35 and 49 samples was also performed as an alternative method. LC–MS/MS followed by MaxQuant (version 1.5.4.0) to search the resulting peptide sequences against UniProt proteomes for *H. sapiens*, *Mus musculus*, and *R. norvegicus* yielded annotations for 13 proteins (Table 1). Raw identification result output by MaxQuant can be found in the supplemental data.

### Retrospective Analysis of MALDI-TOF Protein Profiles Revealed Microproteins of Interest

Manual retrospective analysis was conducted because of the relatively small list of annotated proteins. The dataset reported by Galey *et al.* was reprocessed using MALDIquant, version 1.21 to generate a feature matrix, which was found to contain six of the annotated proteins from LC–MS/MS experiments (highlighted in Table 1) (19). Of these six proteins, two in particular proved to be of interest based on previously reported biological significance in the literature for ovarian cancer and other types of cancer: protein S100-A8 and cystatin A. Protein S100-A8 (*m/z* 10,835 ± 40 ppm, supplemental Fig. S1) was found to be downregulated over time in vaginal lavages as tumor burden increased, whereas cystatin A (*m/z* 11,007 ± 30 ppm) was found to be upregulated over time. Cystatin A was chosen for further analysis and experiments since a protein that is upregulated in the vaginal microenvironment is a preferable biomarker, and replicated upregulation indicates that cystatin A detection is indeed observed with an increase of tumor burden as opposed to being a byproduct of the vaginal lavages. Figure 2 shows the intensity of cystatin A as measured by MALDI-TOF MS. Interestingly, tumor burden as measured by *in vivo* imaging system in mouse 902 appeared to plateau and slightly decrease after day 42 (supplemental Fig. S2). A similar trend was observed in cystatin A intensity for mouse 902 as detected by MALDI-TOF profiling (Fig. 2, *A* and *B*). Although mouse 903 showed no decrease in tumor burden, its cystatin A intensity showed a similar decrease in intensity to that of mouse 902.

### Immunohistochemistry Staining Reveals the Presence of Cystatin A in Murine Reproductive Tissue and Xenografted Tumors

To validate the presence of cystatin A *in vivo*, tumors and reproductive tract tissue were stained for immunohistochemistry (IHC) with antibodies directed against cystatin A (Fig. 3*A*). All the tumors showed varying levels of cystatin A protein expression at the edges of the tumor with a unique “spotting” pattern of distribution; interestingly, this spotting, and therefore cystatin A, was found throughout the tumor collected from mouse 903. Overall, the presence of cystatin A was confirmed in all the tumors, but its localization and abundance lacked consistency. Importantly, OVCAR8 cells are RFP tagged, and no RFP was detected in the lavages indicating that proteins in the lavage are reflective of the tumor microenvironment rather than the tumor cells.

Similarly, IHC staining was also performed on reproductive tissue taken from healthy mice and those with OVCAR8 tumors (ovary, oviduct, uterus, and cervix; Fig. 3*B*). Here, cystatin A can be found in the epithelial cells of the fimbriae of the oviduct and in tissue lining the lumen of the uterus. In oviducts, where OVCAR8 tumors were present, cystatin A abundance was slightly increased, and in the uterus, the cystatin A pattern seen in tumors from Figure 3*A* is also present. In the healthy ovary, cystatin A can be found around the outer surface and “pooling” in the center that lacks ovarian follicles. Finally, the healthy cervix shows large amounts of staining in epithelial tissue. Although the ovaries do not appear to have much cystatin A, differential expression is observed in the other types of reproductive tissue.

### FLOWER Allows for Detection of Low Concentrations of Cystatin A in Limited Biological Samples

FLOWER was also used as an orthogonal method to detect cystatin A. Although other microproteins may have been of interest, the use of FLOWER in the present study was limited to cystatin A since the cost and time required to prepare the microtoroid and acquire specific antibodies for the remaining 12 proteins in Table 1 would prove to be prohibitive. The binding curves for all murine vaginal lavages from days 14, 28, 42, 56, and a 100 pM cystatin A reference standard (positive control) were recorded (supplemental Fig. S3); 10 mM PBS at pH 7.4 was used as a negative control that is not plotted as it is subtracted from the relative shifts to generate FLOWER plots. In mouse 905, there is an increase in cystatin A from day 14 to day 28. In addition, compared with the 100 pM standard, later time points (days 28–56) display equal or greater initial slope values, which proves to be helpful in the quantification and estimation of cystatin A levels and concentrations. To characterize cystatin A levels in each sample, the binding curves were fit with either a linear function,

(1) $$\Delta \lambda_{t}=\text{kt}$$

where *k* is the initial slope and *t* is time, or an exponential growth equation.

(2) $${\Delta \lambda }_{t}=\lambda_{\max}\left(1-e^{-k_{obs}t}\right)$$

where Δ*λ*_*t*_ is the time-dependent resonance wavelength shift and *λ*_*max*_ is the maximum wavelength shift at the plateau (supplemental Fig. S4). The term *k*_obs_ is the observed rate constant for association (20). To obtain the binding rate, the exponential function was differentiated at time *t* = 0, which can be expressed as the initial slope *k* (*k = λ*_*max*_ × *k*_obs_). It should be noted that the initial slope can be calculated to many significant figures; in this study, all reported initial slopes are rounded to three significant figures as that is commonly reported for these data.

To minimize the effect of any variations because of (1) the size, geometry, and characteristics of different microtoroids, (2) flow conditions of the sample, and (3) the immobilization efficiency, which potentially influences the combined dataset, we calibrate all the initial slope values by normalizing the values obtained from the response from a standard 100 pM injection in each sensing trace (21). Here, the initial slope values obtained from the scatter plot and linear regression lines in Figure 4 are plotted as a function of day since tumor implantation for all five mice. Except for mouse 901 and 903, the initial slope values over time show a progressive growth, revealing an overall increase in cystatin A levels (Fig. 4*A*). Mouse 901, however, showed a spike in cystatin A levels on day 14, whereas mouse 903 showed low levels of cystatin A from day 0 to day 42, followed by a sudden increase in the initial slope from −5.676 to 25.157 fm/s at day 56, which is consistent with MS data. The spike in cystatin A in mouse 901 and low concentrations of cystatin A in mouse 903 is most likely attributed to biological variance and/or sampling error. Using the 100 pM cystatin A standard’s initial slope as a reference, cystatin A levels in the diluted vaginal lavages of the remaining four mice are greater than or equal to 100 pM at 42 days post xenograft. Since murine vaginal lavages were diluted a 1000-fold, estimated cystatin A levels are greater than or equal to 100 nM at day 42.

### Cystatin A Detected in Tampon Extracts From Patients Prior to Surgery *via* MALDI-TOF MS Protein Profiling and FLOWER

To determine the feasibility of measuring cystatin A through this workflow from clinical samples, informed consented women were asked to wear a tampon 24 h prior to diagnostic surgery for gynecological cancers. Banked materials were extracted, and the samples were analyzed *via* MALDI-TOF protein profiling. FLOWER was then used as an orthogonal means of detection while also providing an estimated concentration for cystatin A across the samples. Here, six extracted patient samples from the tampons were obtained. Three patient samples were diagnosed as benign growths, whereas the remaining three samples were diagnosed with different histotypes of ovarian cancer. Table 2 contains additional clinical information on each patient sample used in the study.

**Table 2:** Information on patient-derived tampons including primary cancer, clinical diagnoses, and initial slopes and estimated concentration based on FLOWER

ID	Primary cancer	Diagnosis	Patient age	Menopause status	Initial slope (fm/s)	Estimated concentration (pM)
TP10	Benign	Endosalpinogiosis	46	Postmenopausal	−6.267	0.571
TP48	Benign	Leiomyoma	63	Postmenopausal	12.860	713.894
TP50	Benign	Endosalpingiosis	38	Premenopausal	5.318	42.913
TP24	Ovarian	Clear cell carcinoma	65	Postmenopausal	10.510	297.282
TP34	Ovarian	Low-grade serous carcinoma	33	Premenopausal	11.432	358.195
TP42	Ovarian	Carcinosarcoma	59	Postmenopausal	2.426	14.606

Following the protein extraction from a portion of the tampon, 48 technical replicate MALDI-TOF MS protein profiles were collected for each extract. The dataset was then queried for the presence of cystatin A. In the tampon extracts, cystatin A in samples diagnosed with ovarian cancer displayed an overall two-fold increase compared with samples diagnosed as benign when comparing the mean intensities as measured by MALDI-TOF MS (Fig. 5*A*). This observation held true when comparing the median intensities as well. However, it is important to note that there appeared to be variability among technical replicate spectra from the same patient/biological sample. One possible reason for this variability can be variation in cocrystallization during sample preparation, which highlights the importance of sample preparation in this workflow. Furthermore, not all ovarian cancer samples displayed elevated levels of cystatin A (*i.e.*, TP42); similarly, cystatin A levels in benign samples were also variable.

The concentrations of cystatin A in six human tampon samples were measured using FLOWER. Cystatin A levels in each patient-derived tampon extract were characterized based on the initial slopes, *k*, obtained from their associated binding curves (Fig. 5*B*). To further quantify the concentrations of cystatin A, a concentration–response calibration curve was generated by plotting *k versus* concentrations following a linear relation over a range of concentration from 0.1 to 100 nM (supplemental Fig. S5). Table 2 shows the initial slopes and estimated concentration for all six tampon extracts. These concentrations are derived from mapping their corresponding initial slopes onto the calibration plot. FLOWER detected very low levels of cystatin A in TP10. In addition, the highest level of cystatin A was detected in TP48 at a concentration of ∼714 pM.

## Discussion

MALDI-TOF MS protein profiling is a powerful tool that is leveraged in a variety of clinical and academic settings using instruments such as the Biotyper (Bruker Daltonics) and VITEK (bioMerieux) (2). Food and Drug Administration–approved protocols have been in use and allow for high confidence identification of bacteria and yeast (22). Similarly, the Mass-Fix assay has been developed and used clinically to monitor monoclonal proteins (M-proteins) in serum collected from plasma cell dyscrasias patients using MALDI-TOF MS protein profiling (23, 24, 25, 26, 27). Mass-Fix has been applied to multiple myeloma, a cancer that forms from plasma cells and has a low 5-year survival rate (55.6%) (https://seer.cancer.gov/statfacts/html/mulmy.html), (28). By analyzing serum, which is devoid of red blood cells, white blood cells, erythrocytes, and clotting factors, Mass-Fix monitors a relatively simple biofluid. Petukhova *et al.* (9) have previously optimized a method for the detection of microproteins from a more complex biofluid (*i.e.*, whole-cell cultures) in which a mixture of cells were present and analyzed using MALDI-TOF protein profiling (10). A follow-up study by Galey *et al.* used MALDI-TOF MS protein profiling to identify features that are differentially expressed over time as tumor burden increased in murine vaginal lavages. Here, protein profiling was shown to be a viable method for detecting statistically significant features of interest even with increased sample complexity.

Previously, Wei *et al.* (29) have used LC–MS/MS-based bottom–up proteomics to detect protein S100-A6 from serum originating from a murine xenograft model using SKOV3 cells. IHC, *in vivo* bioluminescent imaging, and a modified ELISA assay (ECLISA) were used to show the potential of protein S100A6 as a clinically relevant ovarian cancer biomarker. This report is interesting since in the present study, we have taken a similar approach and detected and annotated several S100 proteins (*i.e.*, S100-A7, S100-A8, S100-A9) in addition to cystatin A (also known as stefin A or acid cysteine proteinase inhibitor) among other microproteins (Table 1; supplementary Information) from murine vaginal lavages containing cells and biomolecules from the vaginal microenvironment. Although cells were present, we have previously shown that fluorescently labeled tumor cells were not detected *via* microscopy or flow cytometry despite their close proximity to the sampling site, indicating the differential expression of microproteins over time were likely the result of changes in the microenvironment and not the OVCAR8 tumors themselves (9). Cystatin A is a human cysteine proteinase inhibitor first identified from the cytosol of human polymorphonuclear granulocytes and has been shown to play roles in various cancers (*i.e.*, colorectal cancer, breast cancer, non–small-cell lung cancer) and diseases (*i.e.*, psoriasis, glaucoma) (30, 31, 32, 33, 34, 35, 36, 37, 38, 39, 40, 41, 42). As type 1 cystatin, cystatin A is normally found intracellularly; however, it has also been shown to appear in biofluids (43, 44). MALDI-TOF MS protein profiling revealed differential expression of cystatin A, which was annotated using LC–MS/MS-based bottom–up proteomics. Lah *et al.* (45) have previously observed upregulation of cystatin A in ascites fluid (buildup of excess fluid in the abdomen), whereas Kastelic *et al.* (46) have observed downregulation of cystatin A in ovarian carcinoma (derived from epithelial tissue). Therefore, the increase of cystatin A in the vaginal microenvironment over time was consistent with data reported by Lah *et al.* as we have also observed upregulation of cystatin A in fluids derived from the vaginal microenvironment (*i.e.*, vaginal lavages). In addition, the downregulation of cystatin A observed in malignant cervical epithelial tissue is consistent with the study by Kastelic *et al.* IHC staining was used to validate the presence of cystatin A in tumors, healthy, and malignant murine reproductive tissues; furthermore, cystatin A appeared to be localized to epithelial cells lining most of the reproductive tract. Interestingly, cystatin A has been found to be localized to the nucleoplasm and cytosol of esophageal and vaginal tissue in humans. Tissue staining images publicly available in the Human Tissue Atlas suggest a similar pattern of cystatin A expression as seen in tissue sample ID T-83000 from patient ID 2004 using cystatin A antibody HPA001031 to that observed in Figure 3, albeit at low concentrations. Expression appeared to be increased in patient-derived tissue diagnosed with cervical cancer in the Human Tissue Atlas, but the level of cystatin A appeared to be dependent on the antibody used.

After observing upregulation of cystatin A in protein profiles over time and validating its presence in tissues, we employed FLOWER as an orthogonal method of detection to explore whether we could measure cystatin A more reliably from earlier time points and determine the limit of detection. We found that FLOWER detected the presence of cystatin A at picomolar concentrations. Early stages of disease are often difficult to detect because of our inability to reliably detect changes in protein and metabolite expression downstream of genetic mutations with current methods (*i.e.*, MALDI-TOF MS and ELISA). In diseases such as ovarian cancer, early screening of disease can be correlated to improved patient prognosis (https://seer.cancer.gov/statfacts/html/ovary.html). Therefore, the development of label-free technologies capable of lower limits of detection for specific analytes is important toward the goal of screening for early stage disease.

Based on the data presented here, the combination of untargeted (MALDI-TOF MS protein profiling) and targeted (FLOWER) detection methods provides a powerful platform that has led to the detection of cystatin A, a promising candidate ovarian cancer biomarker. In addition, we have validated this workflow using clinically relevant samples in the form of protein extracts obtained from tampons worn by patients prior to surgery, proving that the combination of these high-throughput and sensitive detection methods are able to detect and quantify biomarkers at picomolar concentrations from a novel and noninvasive clinical sampling method. As we continue to discover novel biomarkers, they can be used in conjunction with or as a replacement for known biomarkers in multimarker panels. Furthermore, these detection methods can be multiplexed with other current (*i.e.*, biomarker panels) or yet to be developed technologies to further improve our ability to screen for disease in a clinical setting, which opens the potential for larger clinical cohorts and allows for the ability to perform retrospective studies to aid in biomarker discovery.

With that being said, the limitations of the current study must also be considered. (1) Because of the limited nature of the vaginal lavages, sample pooling was required for sufficient material to perform bottom–up proteomics experiments. The collected lavages for individual biological replicates/time points are at most 200 μl in volume of dilute biological fluid–containing cells native to the vaginal microenvironment and extracellular proteins; the low sample volume is a function of the small physical size of the murine vagina. Furthermore, as the microprotein range is our primary target because of its potential compatibility with existing MALDI-TOF MS biotyping instruments, samples were subjected to Amicon spin column filters to enrich the microprotein levels. Therefore, the sample available to perform LC–MS/MS proteomics is inherently not a protein-rich sample; BCA assay results have shown that the protein concentration is below the limit of detection. Therefore, pooling is required to be able to detect peptides above the limit of detection for MS. As observed by Diz *et al.* (47), pooling samples not only can provide benefits including reduction of biological variance in biological replicates but also can result in reduced statistical power and can leave low abundance proteins undetected. Alternative approaches to a longitudinal study to achieve more protein depth may be possible, but they would require mice to be sacrificed at each time point to allow for protein extractions from reproductive organs and tissue, which would vastly increase the number of mice needed. (2) Another limitation of the current study is the lack of tissue to correlate with every lavage time point. (3) Targeted detection *via* FLOWER requires the presence of recombinant monoclonal antibodies specific to the protein of interest on the microtoroid, which may prove difficult for proteins without commercially available antibodies. Furthermore, as seen in data from the Human Tissue Atlas discussed previously, the binding affinity of different antibodies to a protein of interest can vary, which can greatly affect the results produced by FLOWER. (4) Although the mean concentration of cystatin A in patient-derived ovarian cancer samples appeared to be higher than in benign samples, it is important to consider the relatively small sample size used in the current study. As described in Table 2, diagnoses for samples with the same primary cancer were not uniform, which may have affected the variable amounts of cystatin A present in said samples despite being diagnosed with the same primary cancer. Therefore, cystatin A requires detection in many more samples for validation; however, it has the potential to be multiplexed with other biomarkers and forms the basis of investigating vaginal microproteins in cancer progression. While the present study used a specific protocol developed at the University of Kansas Medical Center Cancer Center to yield a limited sample size, the lack of accessible biological samples of this type in any biorepositories highlights an ongoing need to develop standardized protocols for sample collection and storage to allow for studies with greater statistical power. (5) Furthermore, as the samples were deidentified, patient demographic was not taken into consideration during data analysis. (6) Finally, “healthy” controls were not available for comparison, as there were still benign masses removed from the patients that wore those tampons. In the future, inclusion of tampons or lavages from women without any gynecological malignancies may allow us to observe changes in biomarker levels in relation to disease progression. Despite these shortcomings, a major advantage of our study was that lavages and tissues were all collected from the same mice used previously for fingerprinting. We leveraged the limited biological fluids for both identification, validation, and specific sensitive detection which also highlighted the biological variability of this disease in our model organisms. Finally, we were able to validate our findings from our model organism in patient-derived samples to highlight the translational potential of this workflow. We believe the reported workflow has a high potential to achieve a routine screening method for diseases such as ovarian cancer.

## Data Availability

MALDI-TOF MS protein profiles previously reported on by Galey *et al.* can be found in MassIVE repository MSV000083628. Raw data from LC–MS/MS-based bottom–up proteomics and MaxQuant parameters/results can be found in MassIVE repository MSV000088568. MALDI-TOF MS protein profiles for patient-derived tampons can be found in MassIVE repository MSV000090494. All code and data used for MALDI-TOF MS preprocessing and figure creation can be found at https://github.com/gtluu/cystatin_a_figures.

## Supplemental data

This article contains supplemental data (48, 49, 50, 51, 52, 53, 54, 55, 56, 57, 58, 59, 60, 61, 62, 63, 64, 65, 66, 67, 68, 69, 70, 71, 72, 73, 74, 75, 76, 77, 78, 79, 80, 81, 82, 83, 84, 85, 86, 87, 88, 89, 90, 91, 92, 93, 94, 95).

## Conflict of interest

J. S. owns a financial stake in Femtorays Technologies, which develops label-free molecular sensors.
