# Supplementary material for: An Integrated Approach to Protein Discovery and Detection From Complex Biofluids
Source: Mol Cell Proteomics. 2023 Jun 9;22(7):100590. doi: 10.1016/j.mcpro.2023.100590 (PMC10388710; doi:10.1016/j.mcpro.2023.100590)
Supplement: Supplemental Data S4 [file mmc4.docx]

**Supporting Information for**

An integrated approach to protein discovery and detection from complex biofluids

Gordon T. Luu^1^, Chang Ge^2^, Yisha Tang^3^, Kailiang Li^4^, Stephanie M. Cologna^5^, Andrew K. Godwin^6,7,8^, Joanna E. Burdette^4^, Judith Su^2,3^*, Laura M. Sanchez^1,^*

^1^ Department of Chemistry and Biochemistry, University of California Santa Cruz, 1156 High St., Santa Cruz, CA, 95064

^2^ Wyant College of Optical Sciences, University of Arizona, 1630 E. University Blvd., Tucson, AZ, 85721

^3^ Department of Biomedical Engineering, University of Arizona, 1230 N Cherry Avenue, Tucson, AZ, 85721

^4^ Department of Pharmaceutical Sciences, University of Illinois at Chicago, 900 S Ashland Ave, Chicago, IL, 60612

^5^ Department of Chemistry, University of Illinois at Chicago, 845 W. Taylor St., Chicago, IL, 60607

^6^ Department of Pathology and Laboratory Medicine, &

^7^ Kansas Institute for Precision Medicine, University of Kansas Medical Center, 3901 Rainbow Boulevard, Kansas City, KS, 66160

^8^ The University of Kansas Cancer Center, 4001 Rainbow Blvd, Kansas City, KS, 66160

*Judith Su, Laura M. Sanchez.

**Email:** lmsanche@ucsc.edu; judy@optics.arizona.edu

The Role of Various Microproteins Annotated via LC-MS/MS in Ovarian Cancer S3

Figure S1. Intensity and linear regression of mean intensity for protein S100-A8 over time S5

Figure S2. Increase in tumor burden in mice over time S6

Figure S3. FLOWER relative shifts over time S7

Figure S4. Detection of known concentrations of cystatin A via FLOWER S9

Figure S5. Cystatin A calibration curve S9

Figure S6. Linear regression of mean intensities of cystatin A with confidence intervals S10

Figure S7. Linear regression of mean intensities of protein S100-A8 with confidence intervals S11

Figure S8. Linear regression of calibrated initial slopes with confidence intervals S12

Figure S9. Tandem mass spectra for cystatin A peptides S13

Figure S10. Tandem mass spectra for protein S100-A8 peptides S15

Figure S11. Tandem mass spectra for dermcidin peptides S18

Figure S12. Tandem mass spectra for histone H4 peptides S19

Figure S13. Tandem mass spectra for protein S100-A7 peptides S20

Figure S14. Tandem mass spectra for histone H2B1 peptides S21

Figure S15. Tandem mass spectra for protein S100-A9 peptides S22

Figure S16. Tandem mass spectra for fatty acid binding protein 5 peptides S23

Figure S17. Tandem mass spectra for calmodulin-like protein 5 peptides S25

Figure S18. Tandem mass spectra for histone H1.2/H1.3/H1.4 peptides S28

Figure S19. Tandem mass spectra for heat shock protein beta 1 peptides S30

Figure S20. Tandem mass spectra for caspase-14 peptides S31

Figure S21. Tandem mass spectra for ubiquitin-60S ribosomal protein L40 peptides S33

Table S1: Linear model results for cystatin A S34

Table S2: Linear model results for protein S100-A8 S34

References S35

**Supplemental Information**

*The Role of Various Microproteins Annotated via LC-MS/MS in Ovarian Cancer*

The S100 family of proteins, containing 24 members, have been well studied and exhibit a host of intracellular and extracellular functions [(49)](https://paperpile.com/c/NFdQ96/38Ktv). Among this family, protein S100-A7, S100-A8, and S100-A9 were annotated from our LC-MS/MS dataset, and the latter two were also found via MALDI protein profiling; these data implicate these proteins in playing a role in ovarian cancer progression and metastasis. Furthermore, the potential of protein S100-A6 as an ovarian cancer biomarker has previously been evaluated using a similar workflow utilizing LC-MS/MS based bottom-up proteomics and a modified ELISA assay (ECLISA) [(50)](https://paperpile.com/c/NFdQ96/Zp8Jw). Bai *et al*. have also evaluated the prognostic value of the protein S100 family as a whole through measuring mRNA expression [(51)](https://paperpile.com/c/NFdQ96/5N3kk). Their results indicate that expression of protein S100-A7, S100-A8, and S100-A9 mRNA in ovarian cancer patients saw minor downregulation, which is in agreement with expression of protein S100-A8 as detected by MALDI protein profiling (**Figure S1**). However, downregulation of these proteins due to ovarian cancer presents a challenge in their utility as a screening biomarker, as their absence in protein profiles is not evidence of their absence in patient derived biosamples.

Fatty acid-binding protein 5 (FABP5) is a fatty acid transporter that has been implicated in a variety of cancer [(52)](https://paperpile.com/c/NFdQ96/DcFEr). In the context of ovarian cancer, the FABP family of proteins have been found to play a part in metastasis. Specifically, FABP4 has been found to be upregulated, resulting in poor patient prognosis [(53–55)](https://paperpile.com/c/NFdQ96/dxUrn+DWz9R+9qkJH). Due to FABPs linkage to poor patient prognosis and apparent upregulation, FABP5 remains a potential screening target in ovarian cancer, which is further supported by its detection via MALDI protein profiling. Furthermore, recent studies have shown that inhibition of lipid transporters causes a reduction of cell growth, cell cycle arrest, and apoptosis [(56)](https://paperpile.com/c/NFdQ96/b17Jx). This indicates that FABPs such as FABP5 are also a potential therapeutic target for the treatment of ovarian cancer and other cancers.

The caspase family of proteins are proteases apoptosis and inflammation, with caspase-14 being unique due to its association with epithelial cell differentiation (i.e. cornification) as opposed to the apoptosis and inflammation [(57, 58)](https://paperpile.com/c/NFdQ96/EGyyr+OnPMS). This indicates that caspase-14 has a large role in epithelial tissue cell death and therefore can play a role in epithelial malignancies. In gynecological cancers such as ovarian cancer, cervical cancer, and cancer of the vulva, decrease in caspase-14 expression has been found to be associated with advanced stages of these cancers [(57, 59)](https://paperpile.com/c/NFdQ96/EGyyr+eKG5L). Although annotated in our LC-MS/MS dataset, this feature was not identified via MALDI protein profiling, indicating that caspase-14 is either below our current limit of detection via MALDI or does not ionize sufficiently.

Calmodulins are messenger proteins that are targeted and activated by Ca^2+^ binding, which leads to activation of downstream signaling pathways. Calmodulin-like protein 5 is a protein from this family that is found in the epidermis. These proteins have been found to be involved in cell migration, invasion, and tumor metastasis [(60)](https://paperpile.com/c/NFdQ96/D46dA). While not found via MALDI protein profiling, calmodulin-like protein 5 was annotated via LC-MS/MS. Kitazawa *et al*. have found that calmodulin-like protein 5 acts as a tumor suppressor gene and is downregulated with the progression of uterine cervical cancer [(61)](https://paperpile.com/c/NFdQ96/WhpLK). Conversely, the related protein Ca^2+^/calmodulin dependent protein kinase kinase 2 (CaMMK2 or CaMMKII) has been found to be upregulated in ovarian cancer cell and ovarian carcinoma [(62, 63)](https://paperpile.com/c/NFdQ96/NsARt+QRbVd). Furthermore, suppression of CaMMK2 has been found to inhibit growth of human ovarian cancer cells *in vitro* [(64)](https://paperpile.com/c/NFdQ96/Dq4d).

Dermcidin is a human derived antimicrobial peptide that plays a role in the host innate immune system. Previous studies have shown that the dermcidin derived peptide Y-30 has been found to play a role in tumorigenesis and cancer progression [(65, 66)](https://paperpile.com/c/NFdQ96/Zx9cx+9RQdF). Overexpression of dermcidin itself has also been found to play a role in various cancers [(67, 68)](https://paperpile.com/c/NFdQ96/y5w63+PUUbV). Its cleaved form, dermcidin-1, has been found in the placenta, but the function of these peptides in the vaginal microenvironment remains unclear[(65, 69)](https://paperpile.com/c/NFdQ96/Zx9cx+5j9qe). However, dermcidin has been hypothesized to induce autophagy in melanoma cell lines when overexpressed as a result of treatment with the natural product seriniquinone [(70)](https://paperpile.com/c/NFdQ96/PLj8A). Furthermore, treatment of ovarian cancer cells (SKOV3) with protein aggregate magnesium-ammonium phospholinoleate-palmitoleate anhydride (P-MAPA) and human recombinant interleukin-12 (hrIL-12) resulted in the upregulation of dermcidin by nearly two-fold compared to the control group [(71)](https://paperpile.com/c/NFdQ96/ROnJO). While not found via MALDI protein profiling, annotation via LC-MS/MS may warrant further investigation in the context of ovarian cancer.

Heat shock proteins (HSPs) are a family of proteins that allow cells to respond to environmental stress, including but not limited to extreme heat, cold, and UV light [(72–74)](https://paperpile.com/c/NFdQ96/OC0aT+fJw4u+0ZetU). The roles of various HSPs in ovarian cancer have been explored [(75, 76)](https://paperpile.com/c/NFdQ96/MYgZF+cQLRK). Heat shock protein beta 1 (HSPB1), also known as HSP27, has been shown to play a role in various cancers including gynecological cancers such as ovarian and endometrial cancer [(75, 76–81)](https://paperpile.com/c/NFdQ96/MYgZF+EbitS+0mQvW+NMM92+QD2VS+QprLz). Hoter and Naim have noted that previous reports have shown HSPB1 to be upregulated in advanced ovarian cancer stages [(75, 76, 82)](https://paperpile.com/c/NFdQ96/MYgZF+cQLRK+u5u7y). Furthermore, several studies have also reported the potential of HSPB1 as a therapeutic target [(77, 83, 84)](https://paperpile.com/c/NFdQ96/5yaLQ+EbitS+MJQ3G). Presence in murine vaginal lavages combined with upregulation indicate that this is potentially a biomarker of interest to be used in future multiplexed ovarian cancer screening efforts as noted by Zhao *et al*. [(85)](https://paperpile.com/c/NFdQ96/VvZnG).

The role of histones and epigenetics in ovarian cancer and other gynecological cancers has been widely reported [(86–90)](https://paperpile.com/c/NFdQ96/EXo85+nA57l+lI2Kv+QTfw8+0ah9r). Epigenetic modifications can result in a broad variety of changes to protein expression that can contribute to the formation and progression of ovarian cancer. Here, histones H1.2, H1.3, H1.4, H2B1, and H4 were annotated from LC-MS/MS data. Quantitative reverse transcription-polymerase chain reaction (RT-PCR) previously revealed that histone H1.3 was upregulated in human epithelial ovarian tumors, while histone 1.4 was found to be downregulated [(86)](https://paperpile.com/c/NFdQ96/EXo85). These H1 linker histones have been found to be important in higher order chromatin folding. Histone H2B has been shown to be susceptible to various modifications, including monoubiquitination; this histone H2Bub1 was found to be downregulated in ovarian cancer [(87, 91, 92)](https://paperpile.com/c/NFdQ96/nA57l+zmFt9+kwCTt). One post-translational modification of histone H4 is acetylation at K16 [(88)](https://paperpile.com/c/NFdQ96/lI2Kv). hMOF has been found to cause this acetylation, and downregulation of hMOF and loss of acetylation at histone H4K16 has been linked to the incidence of ovarian epithelial cancer issues [(93–87)](https://paperpile.com/c/NFdQ96/yc8i7+KsnJz+DKSbQ+nwsKh+vYWjX). Although their role is variable in ovarian cancer, inclusion of upregulated histones may be warranted in multiplexed biomarker panels.


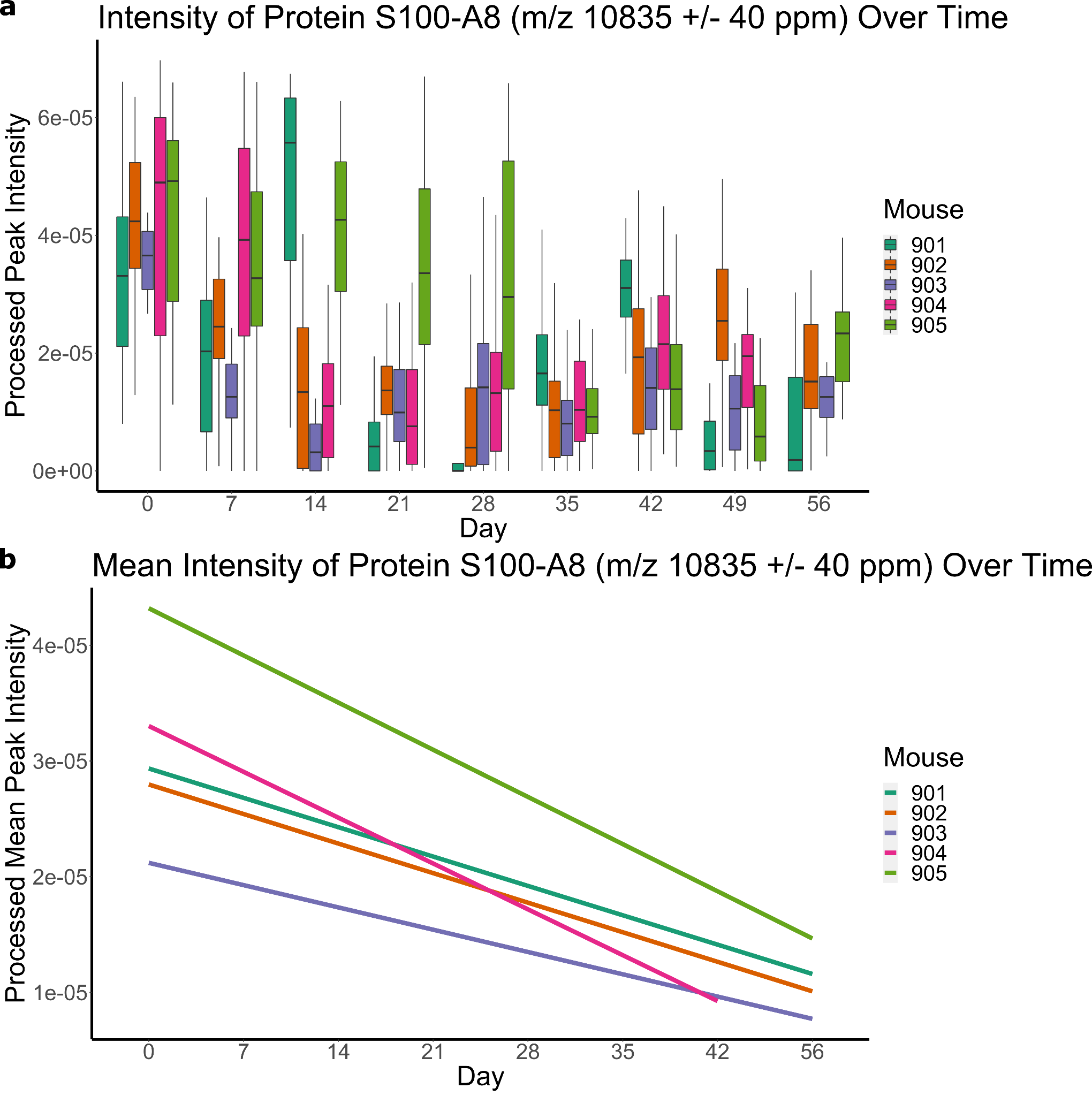


**Figure S1**. (**a**) Box plot showing the intensity of protein S100-A8 at each corresponding time point. (**b**) Linear regression trend lines for the mean intensities of protein S100-A8 at each time point showing downregulation.


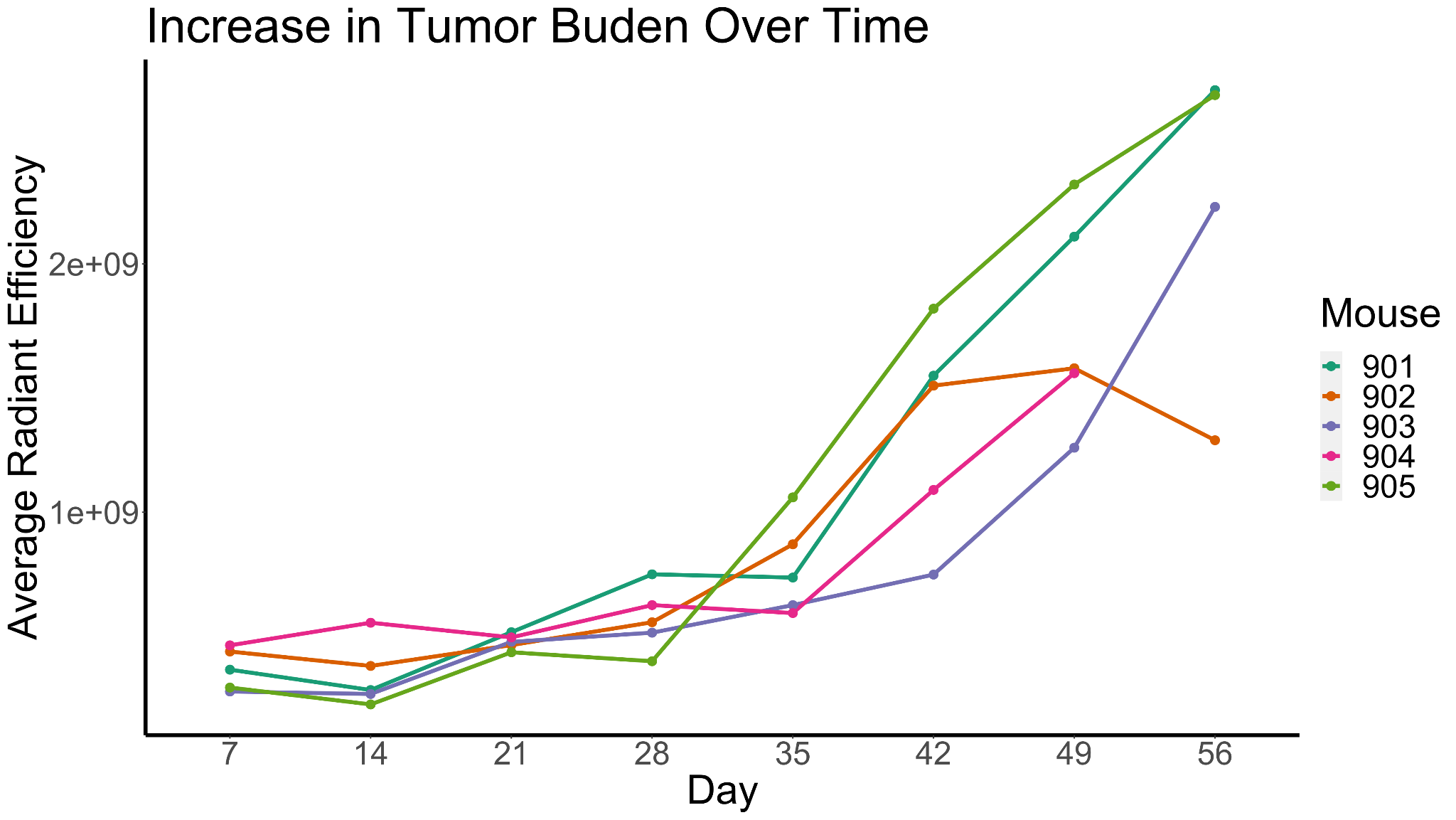


**Figure S2**. Tumor burden in five biological replicate xenograft murine models transfected with OVCAR-8-RFP tumors. Tumor burden was measured weekly over 56 days by IVIS. Here, day 56 was chosen as a humane endpoint as it was far along enough to represent late stage disease.


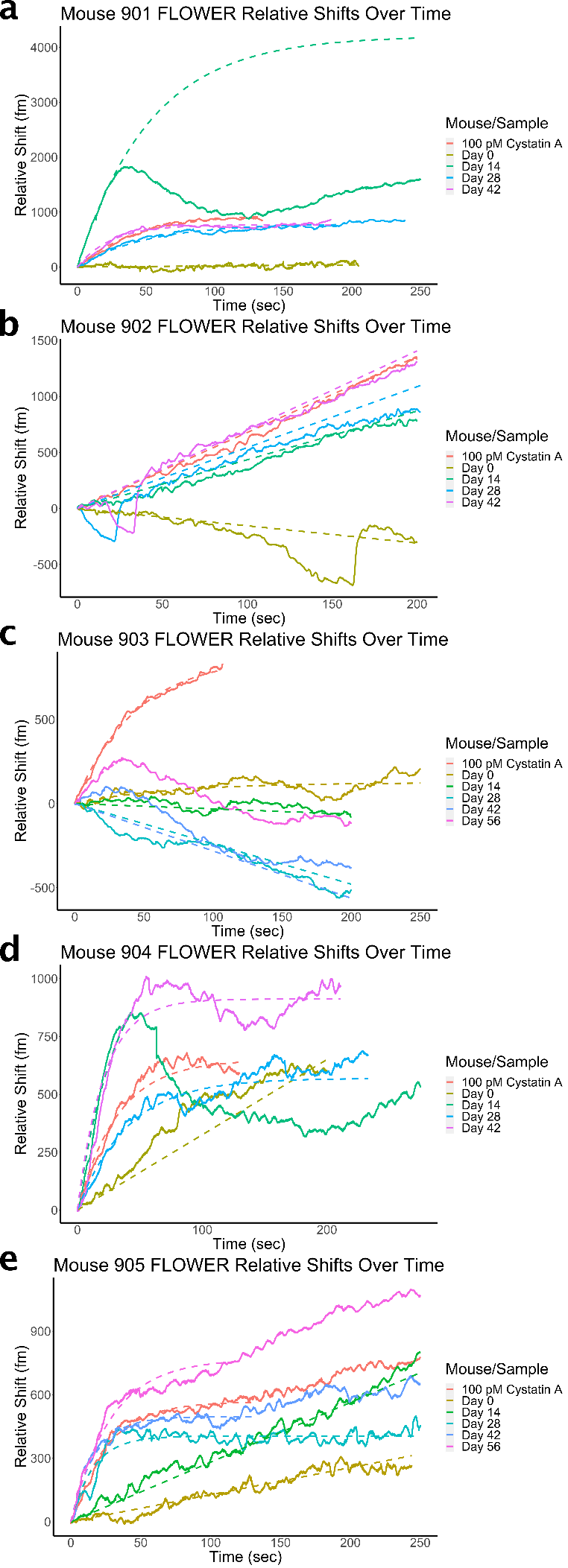


**Figure S3**. Label-free detection of cystatin A using a microtoroid resonator. (**a-e**) Cystatin A binding curves for Mouse 901-905. Like MS protein profiles, Mouse 903 displayed a strange lack of detectable cystatin A. Several time points (*i.e.,* Mouse 901 day 14 and Mouse 904 day 14) also appeared to have abnormal binding curves, most likely owed to biological variability and/or murine model behavior during collection of murine vaginal lavages. The dashed curves represent fits to the experimental data using Equation (2) unless the r-squared value with a fit to Equation (1) was better in which case Equation (1) was used, which has fewer fitting parameters than Equation (2). In both cases, the fit was used to obtain the initial slope of the curve.

**
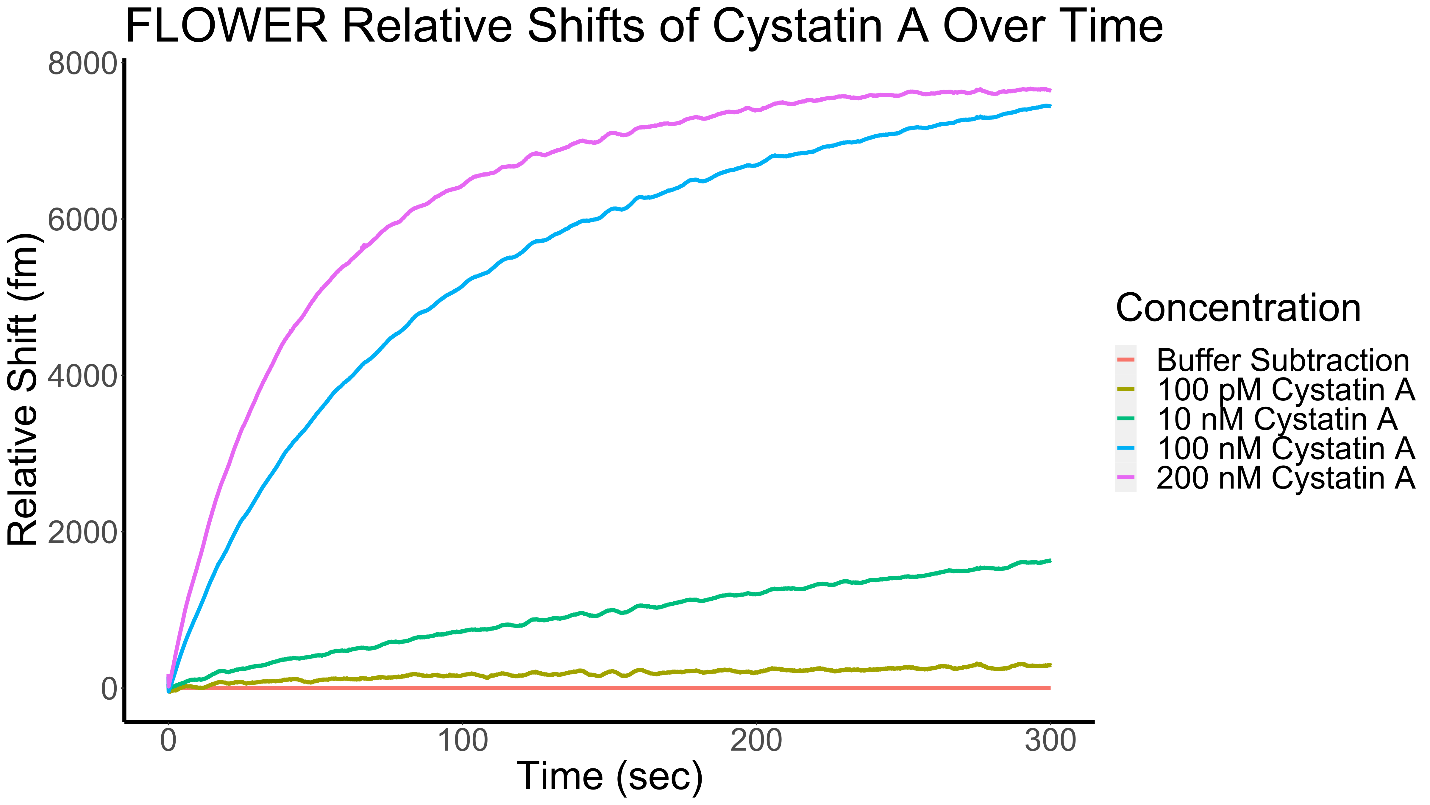
**

**Figure S4**. Detection of known concentrations of cystatin-A binding to anti-cystatin-A using FLOWER. Experiments were performed in PBS.


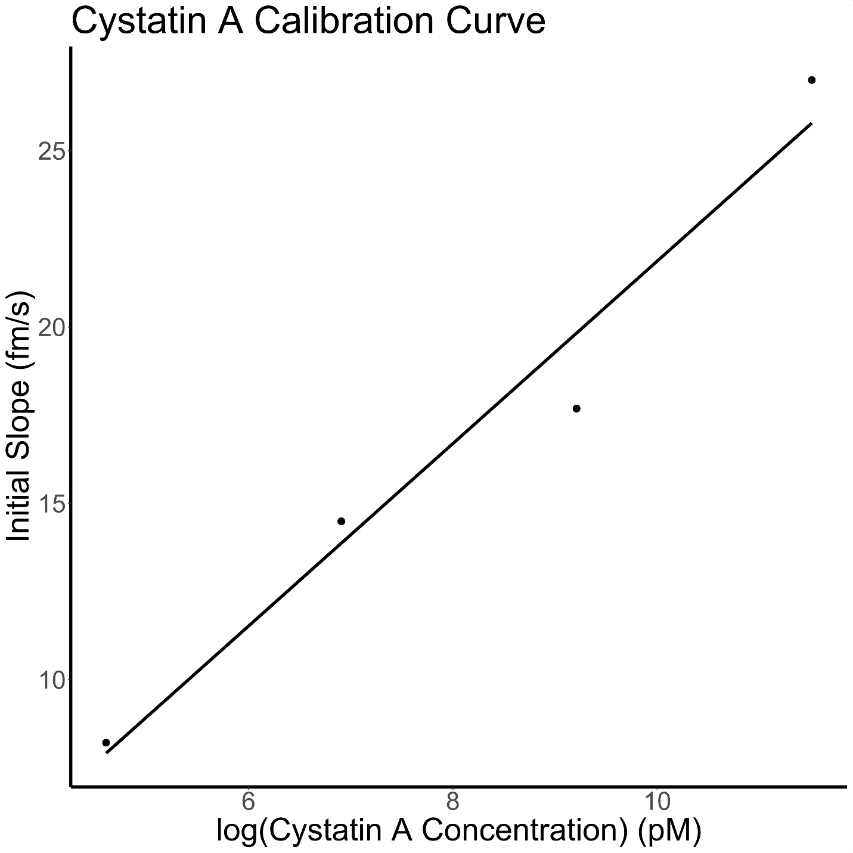


**Figure S5**. Calibration curve for cystatin A displays a linear relationship between the concentration and initial slope as measured by FLOWER.


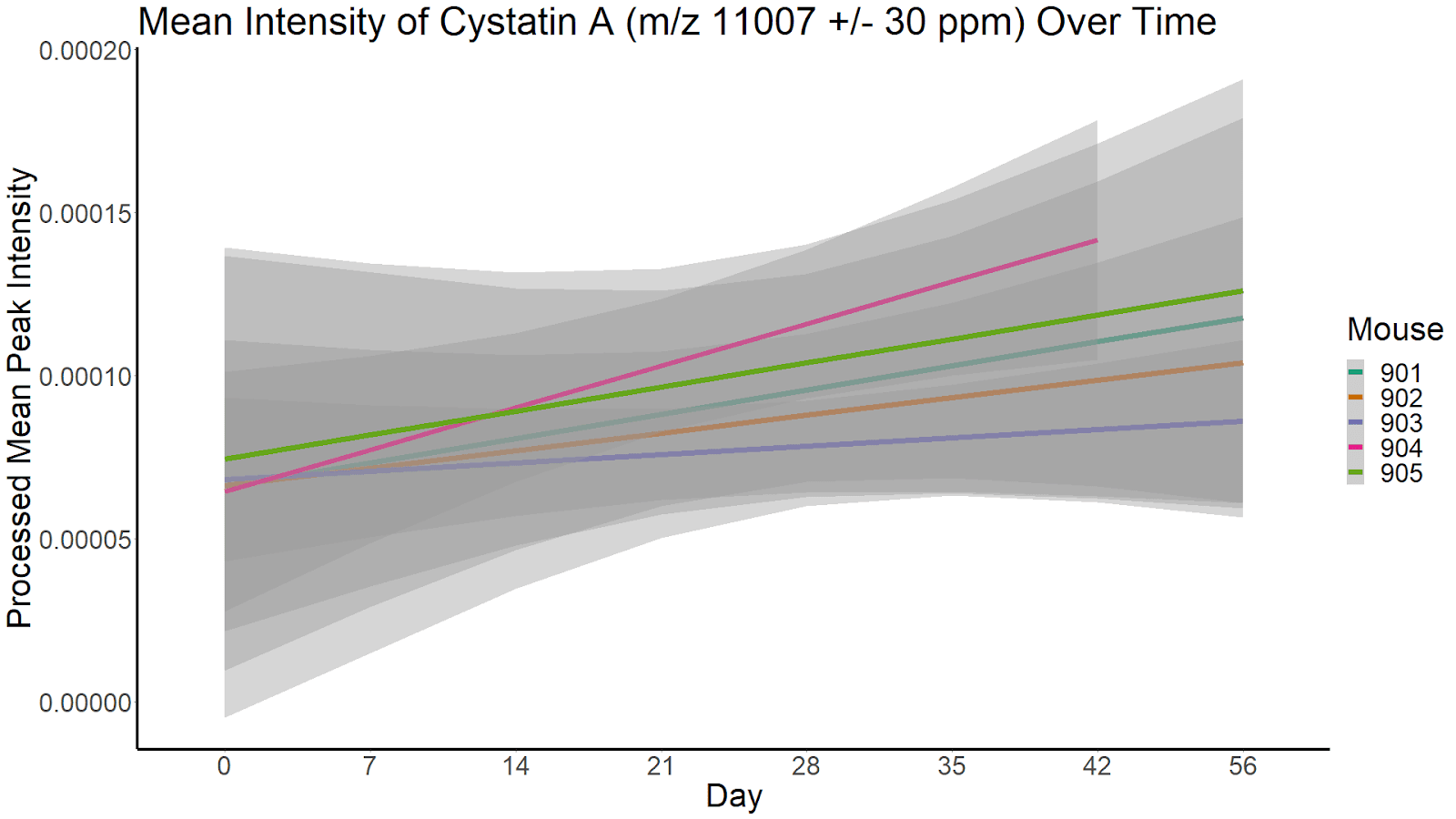


**Figure S6**. Linear regression trend lines for the mean intensities of cystatin A at each time point showing upregulation as plotted in **Figure 2b** with the confidence intervals. Here, expression of cystatin A is similar in each of the five mice and the same clear upward trend (indicating upregulation) over time can be seen as indicated by the overlap in confidence intervals, despite any biological variance that may be present.


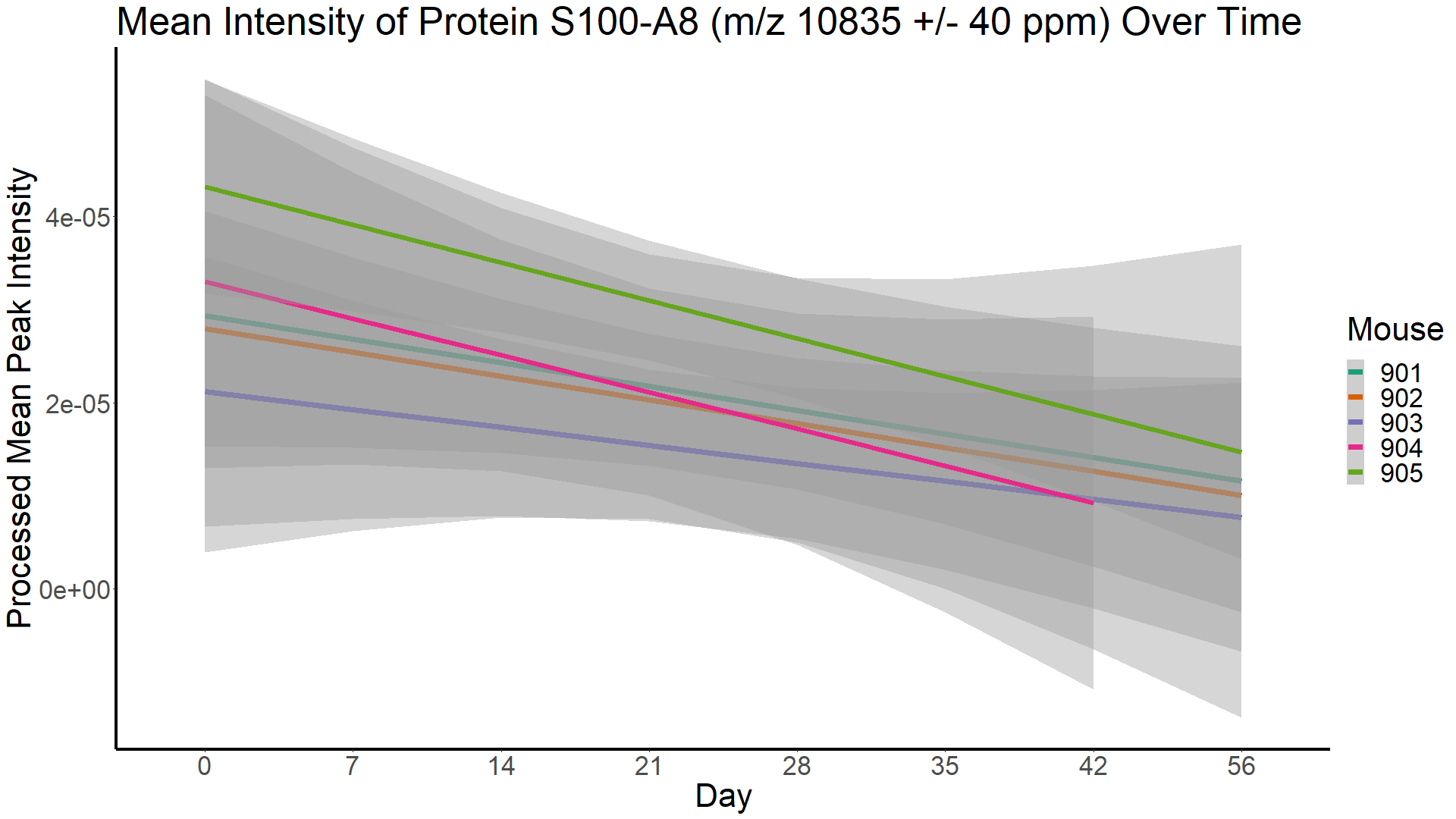


**Figure S7**. Linear regression trend lines for the mean intensities of protein S100-A8 at each time point showing upregulation as plotted in **Figure S1b** with the confidence intervals. Here, expression of protein S100-A8 is similar in each of the five mice and the same clear downward trend (indicating downregulation) over time can be seen as indicated by the overlap in confidence intervals, despite any biological variance that may be present.


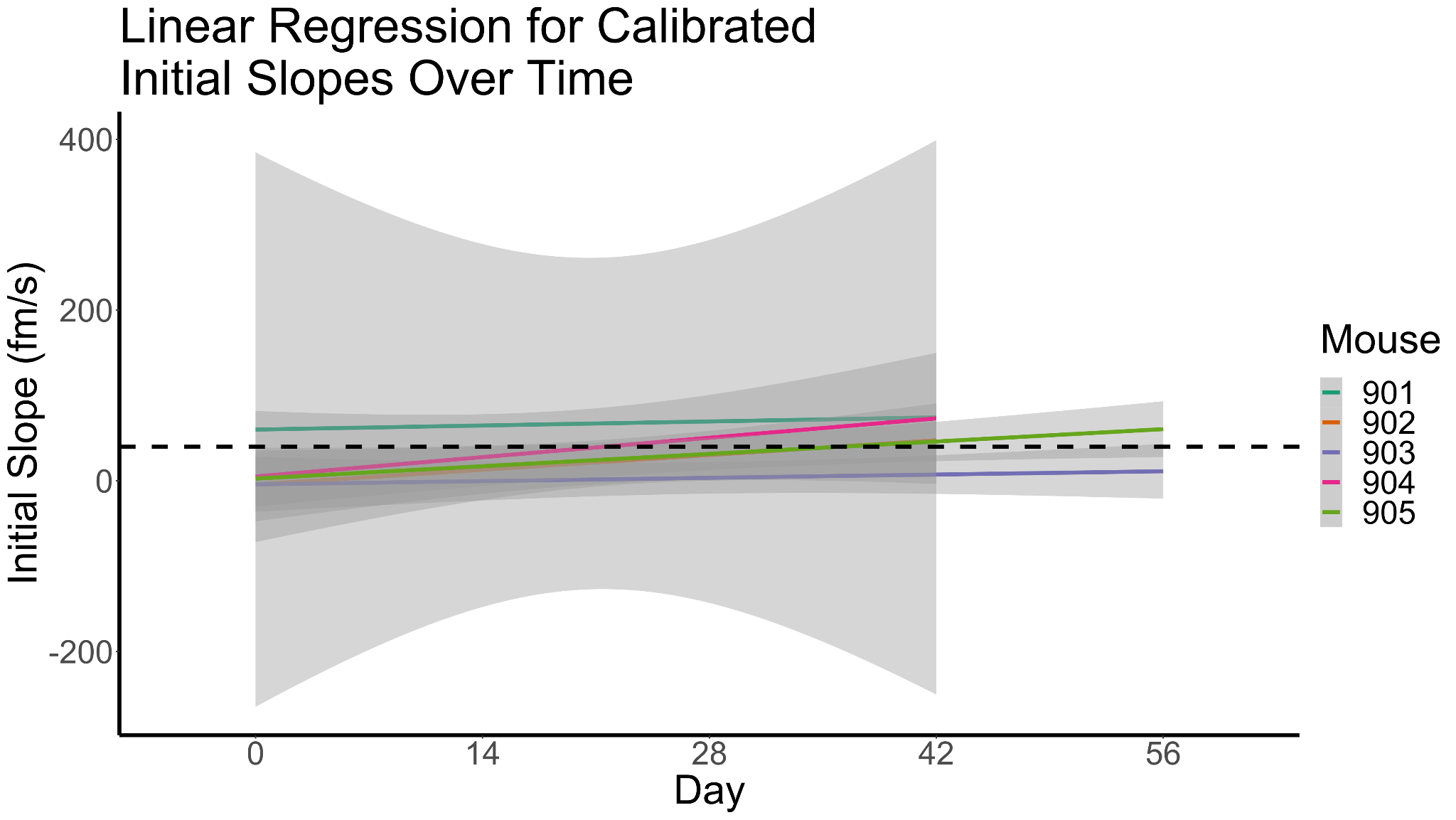


**Figure S8**. Linear regression lines for calibrated initial slopes as determined by FLOWER over time showing upregulation as plotted in **Figure 4b** with the confidence intervals. It should be noted that the large confidence interval from Mouse 901 causes each line to appear linear when in fact there is a clear upward trend as seen in **Figure 4b**.


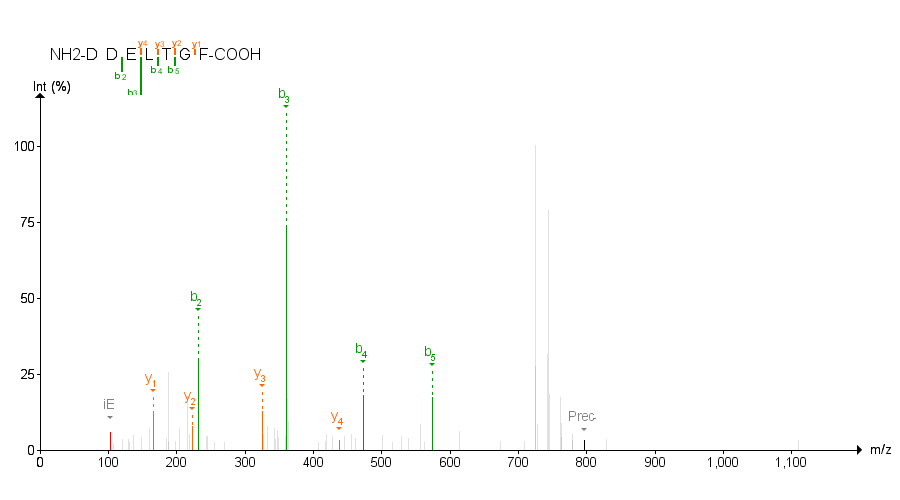


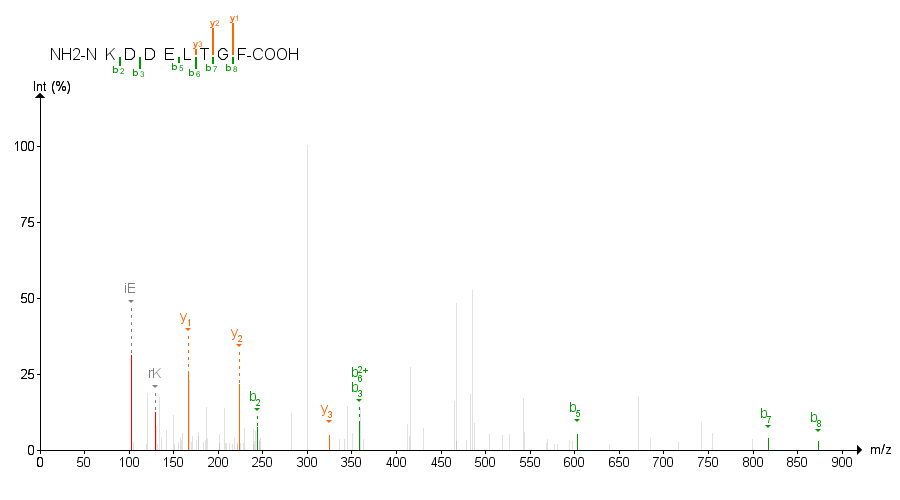


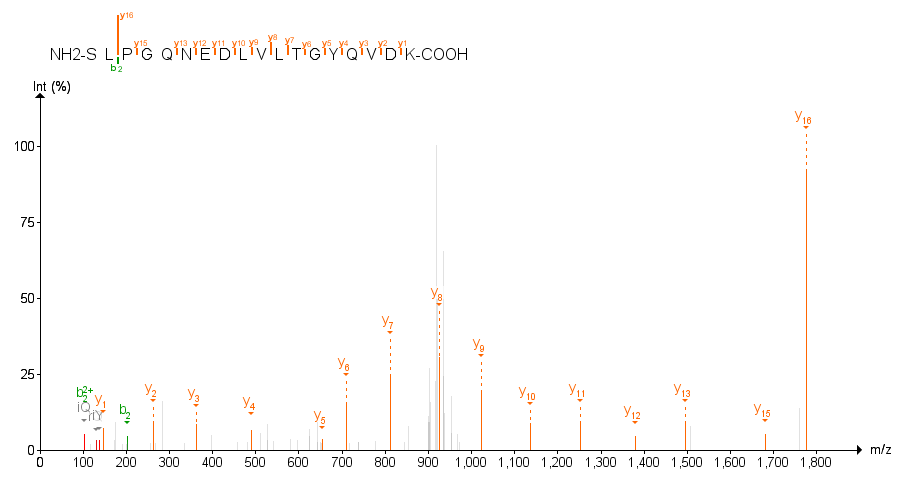


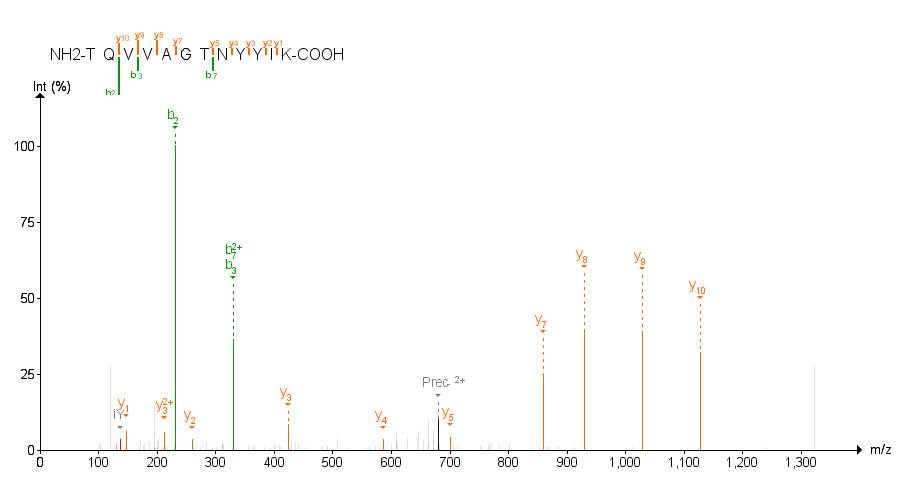


**Figure S9**. Tandem mass spectra for cystatin A peptides annotated via MaxQuant version 1.5.4.0.


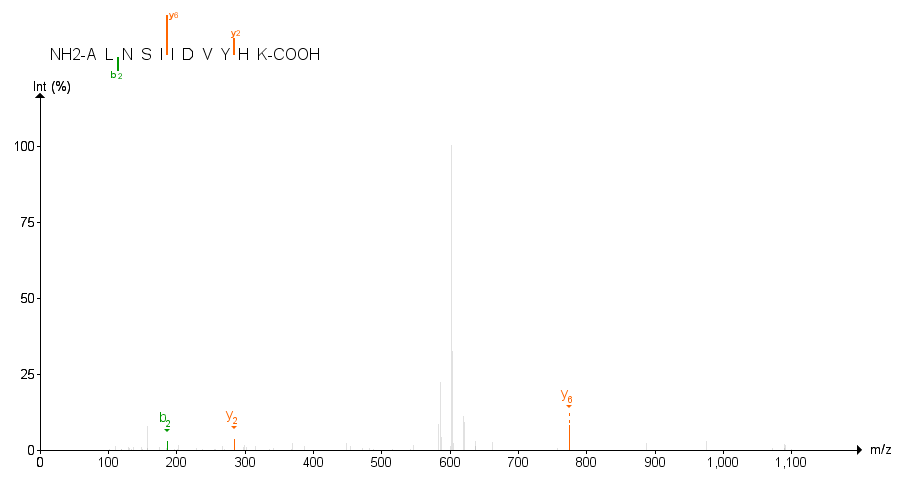


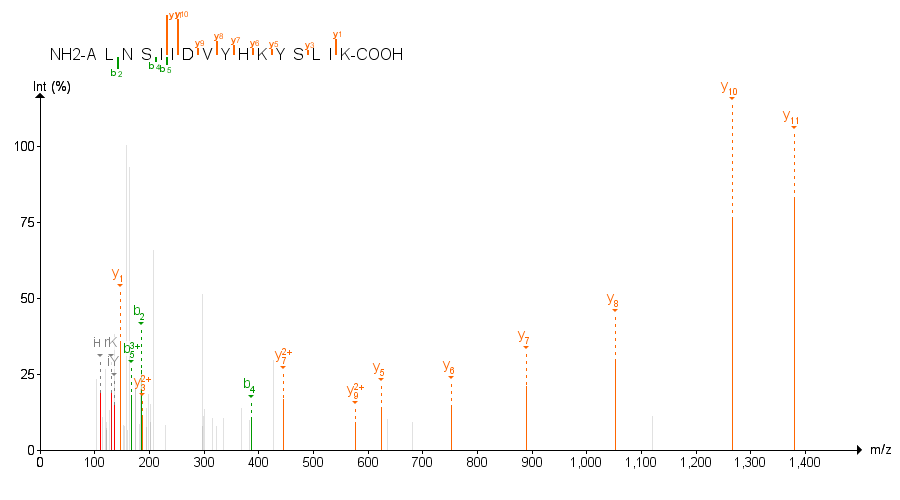


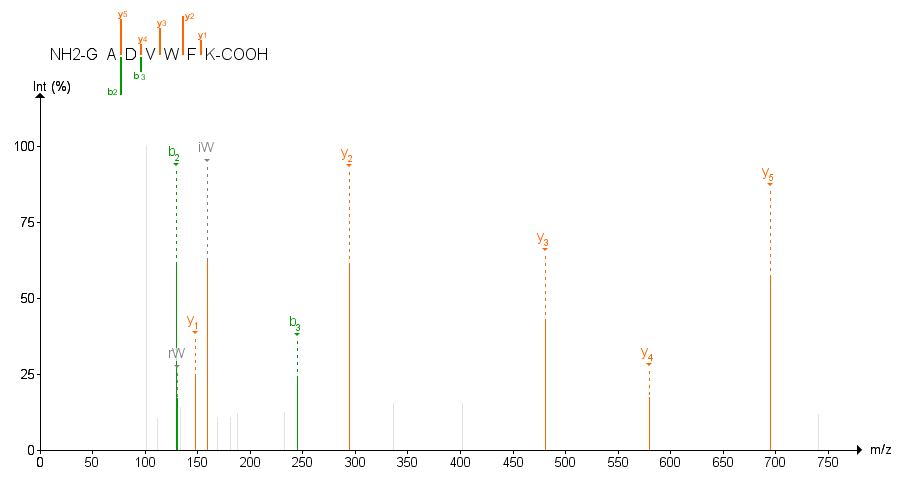


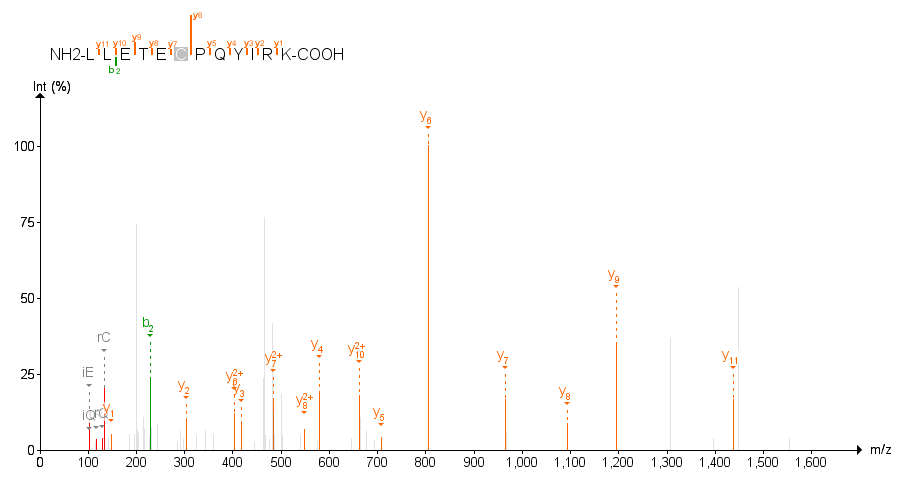


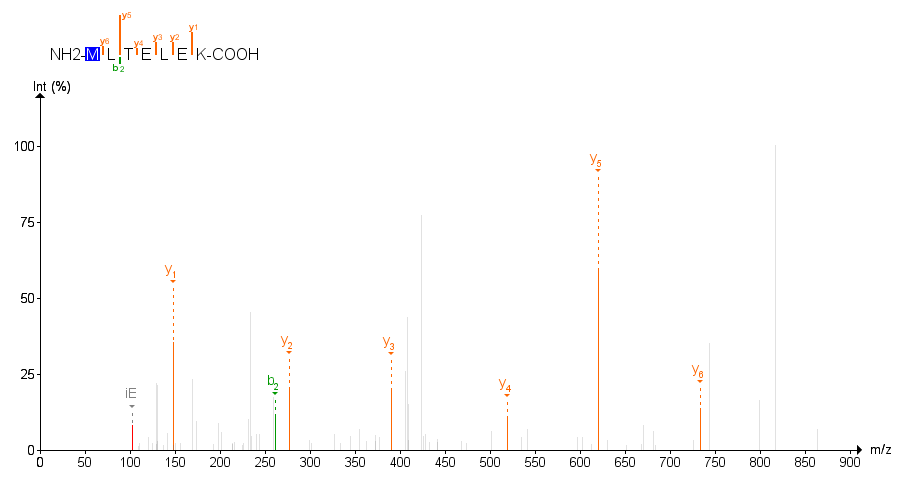


**Figure S10**. Tandem mass spectra for protein S100-A8 peptides annotated via MaxQuant version 1.5.4.0.


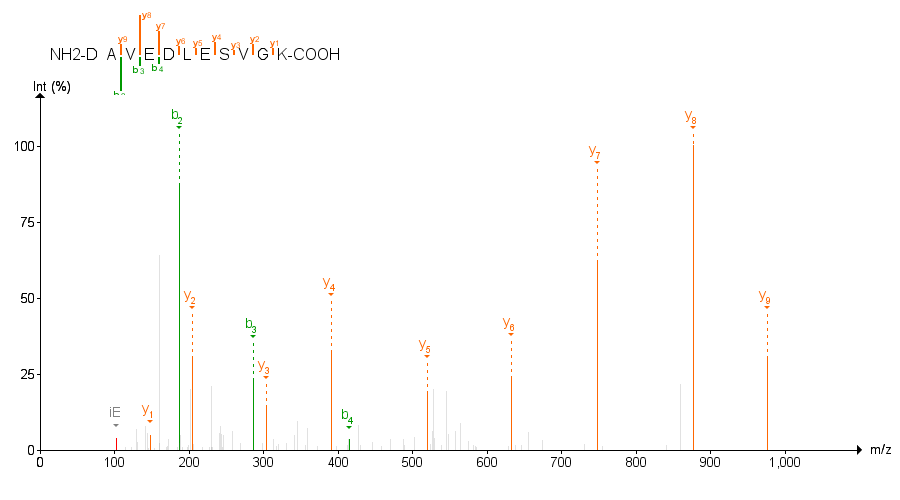


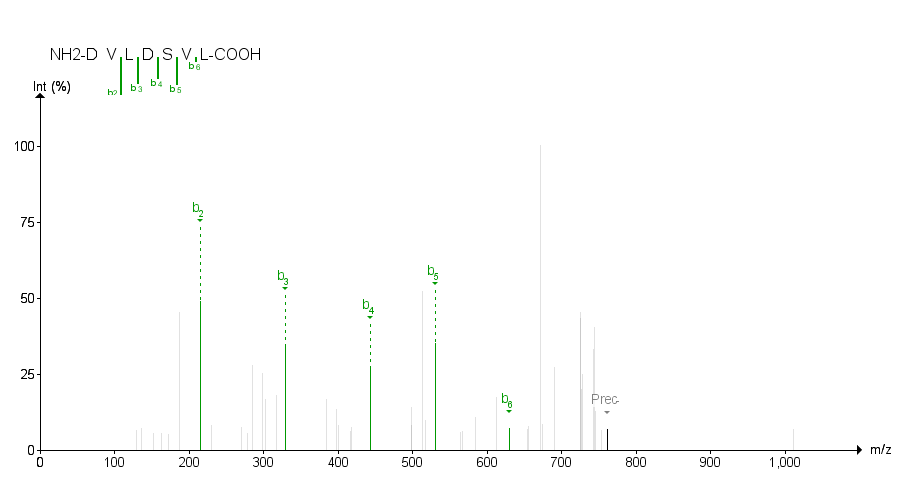


**Figure S11**. Tandem mass spectra for dermcidin peptides annotated via MaxQuant version 1.5.4.0.


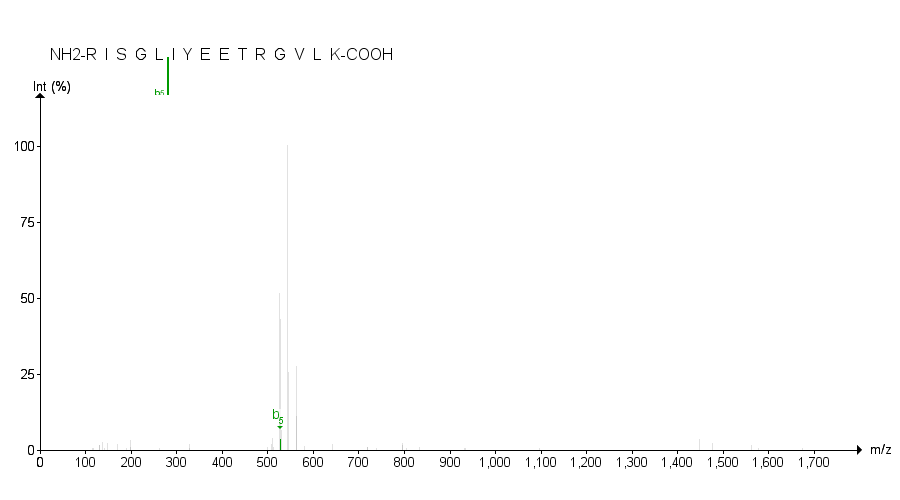


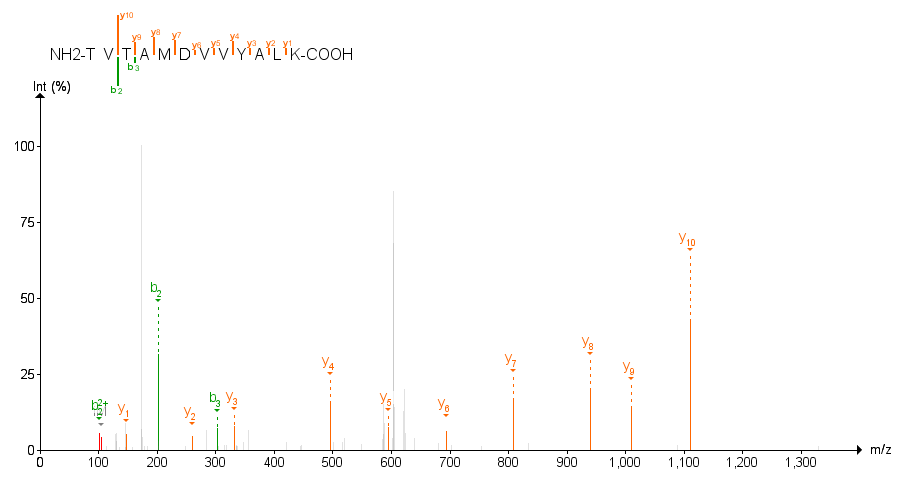


**Figure S12**. Tandem mass spectra for histone H4 peptides annotated via MaxQuant version 1.5.4.0.


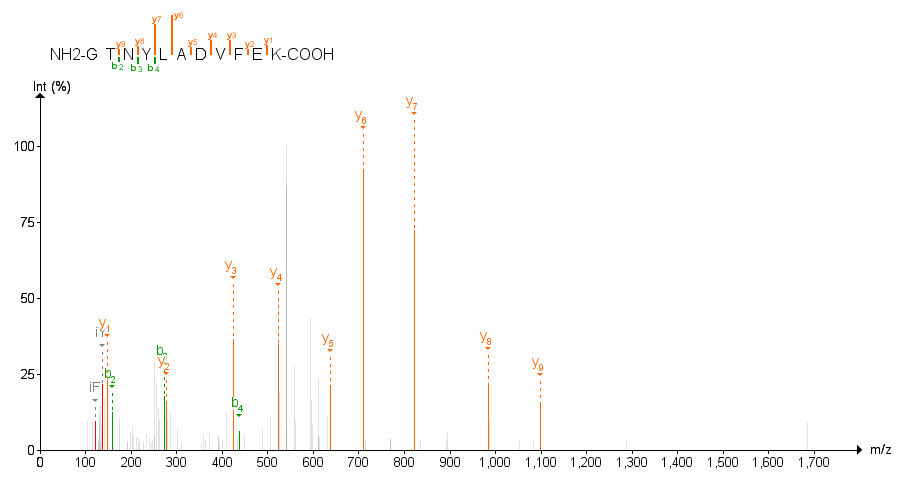


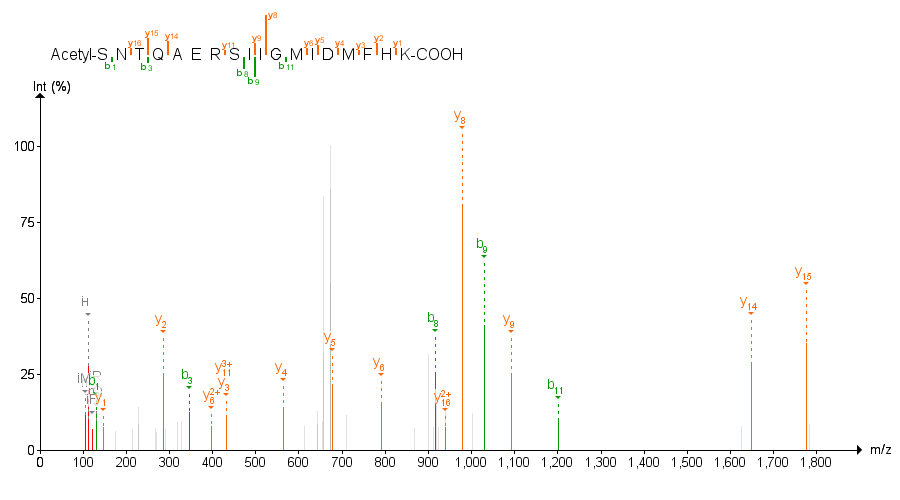


**Figure S13**. Tandem mass spectra for protein S100-A7 peptides annotated via MaxQuant version 1.5.4.0.


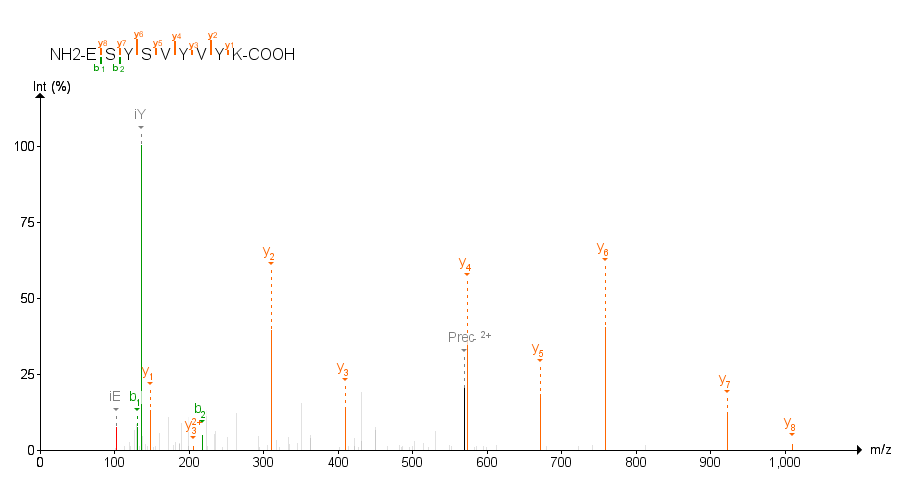


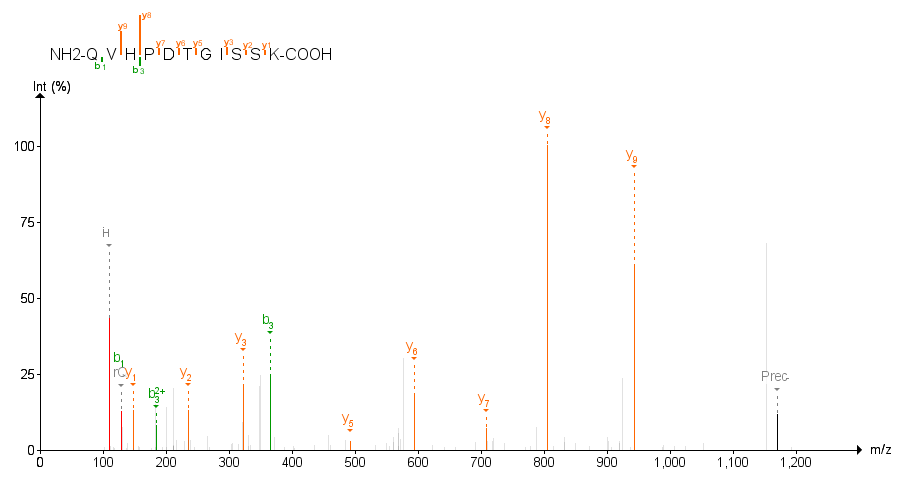


**Figure S14**. Tandem mass spectra for histone H2B1 peptides annotated via MaxQuant version 1.5.4.0.


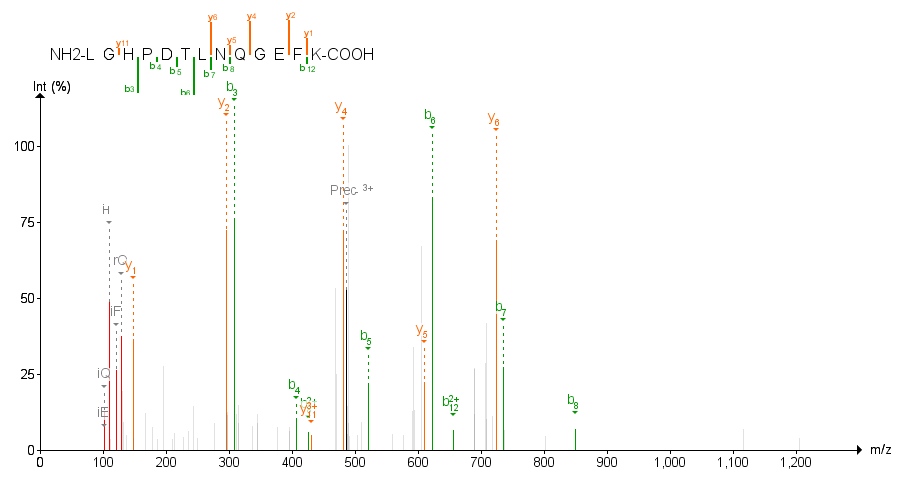


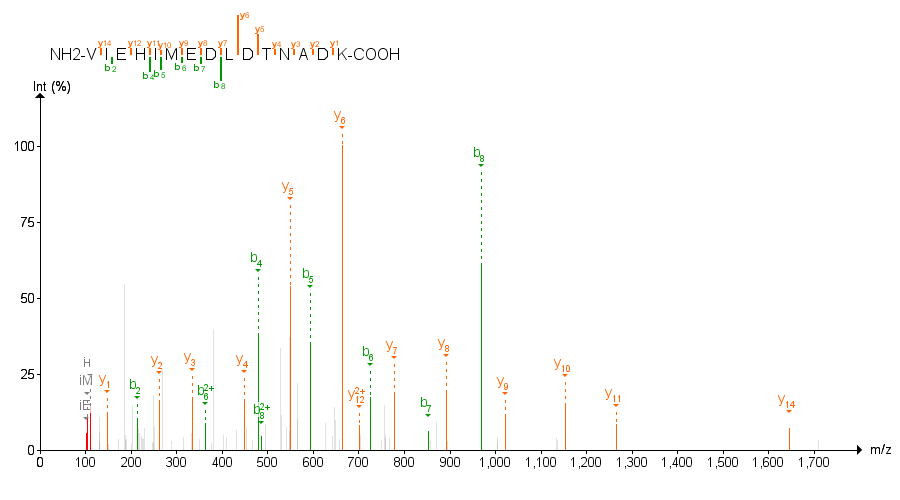


**Figure S15**. Tandem mass spectra for protein S100-A9 peptides annotated via MaxQuant version 1.5.4.0.


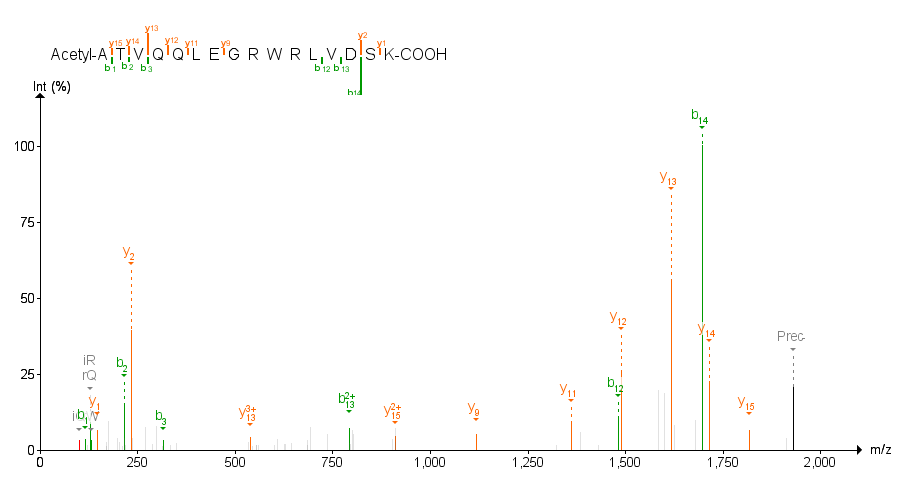


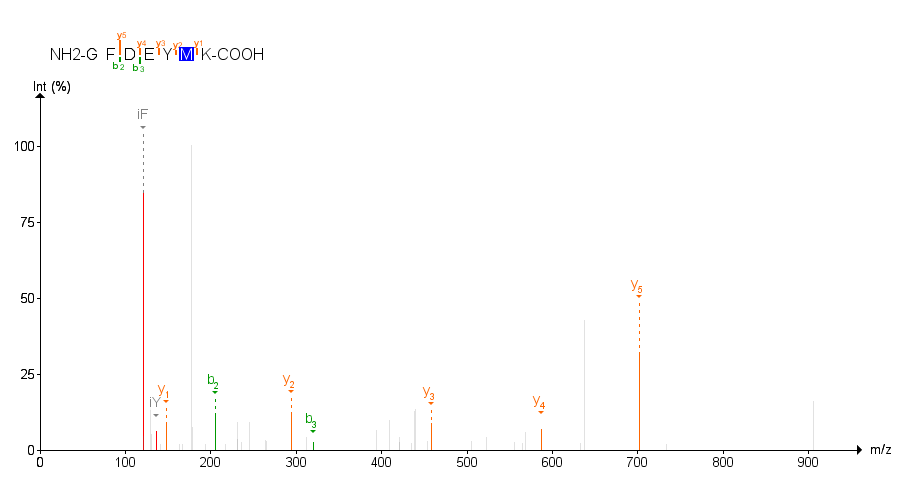


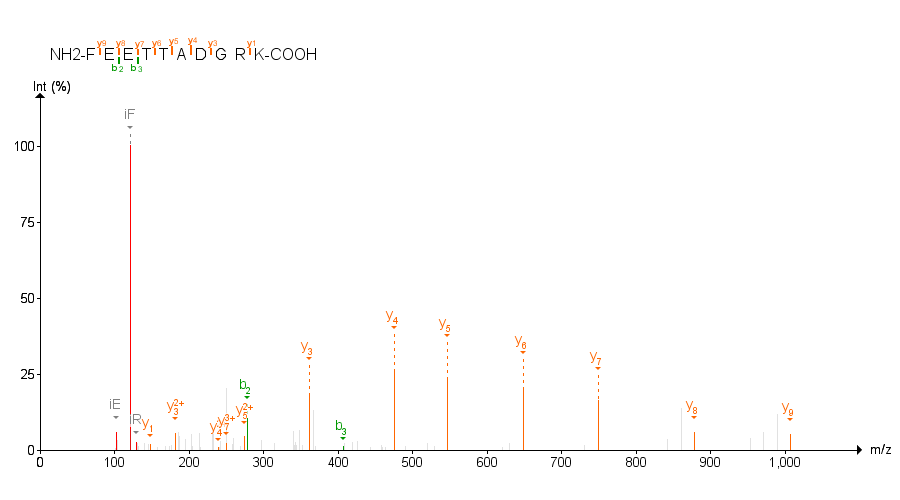


**Figure S16**. Tandem mass spectra for fatty acid binding protein 5 peptides annotated via MaxQuant version 1.5.4.0.


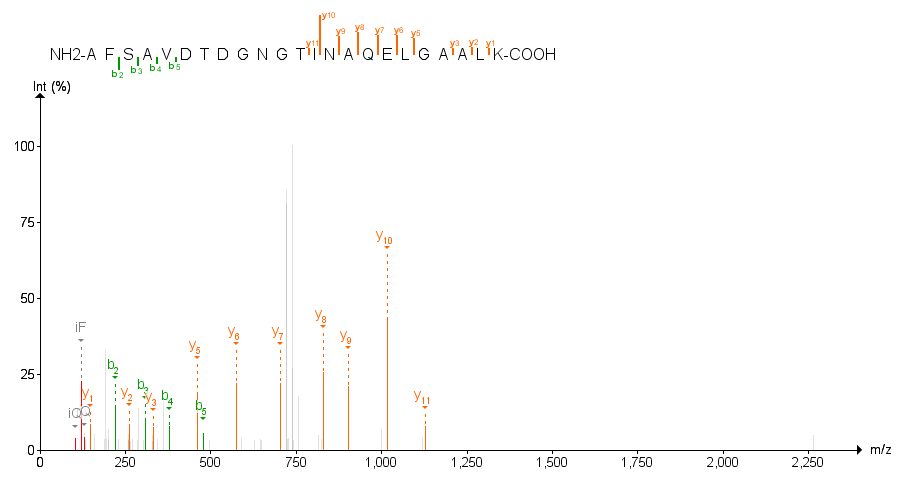


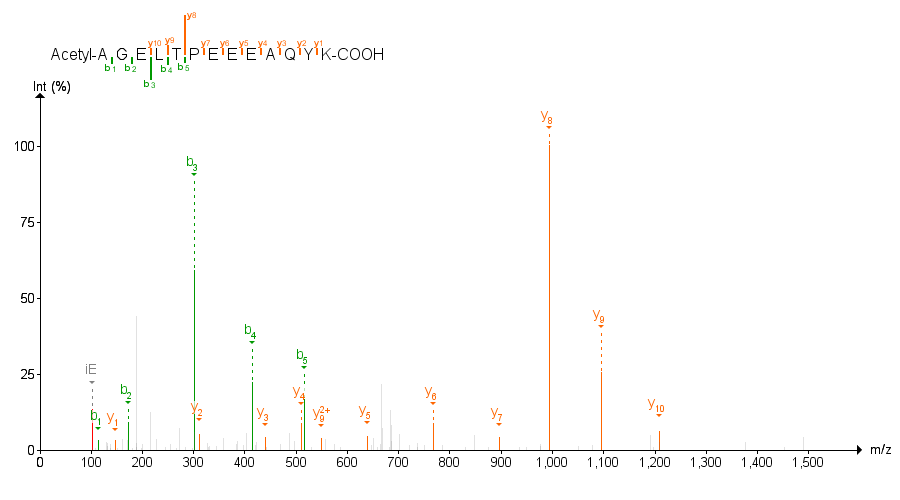


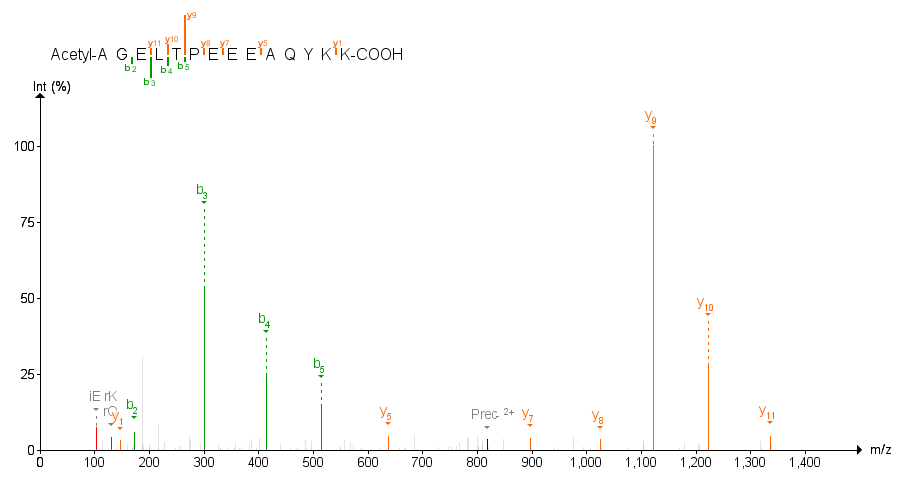


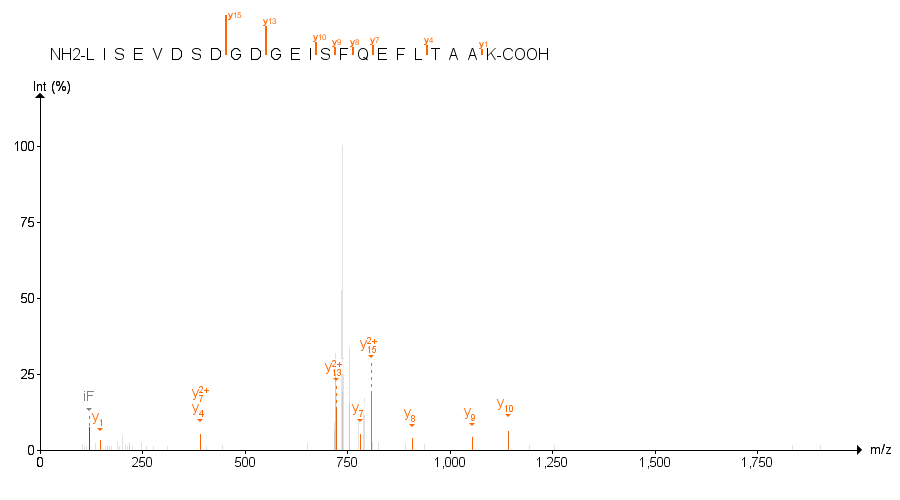


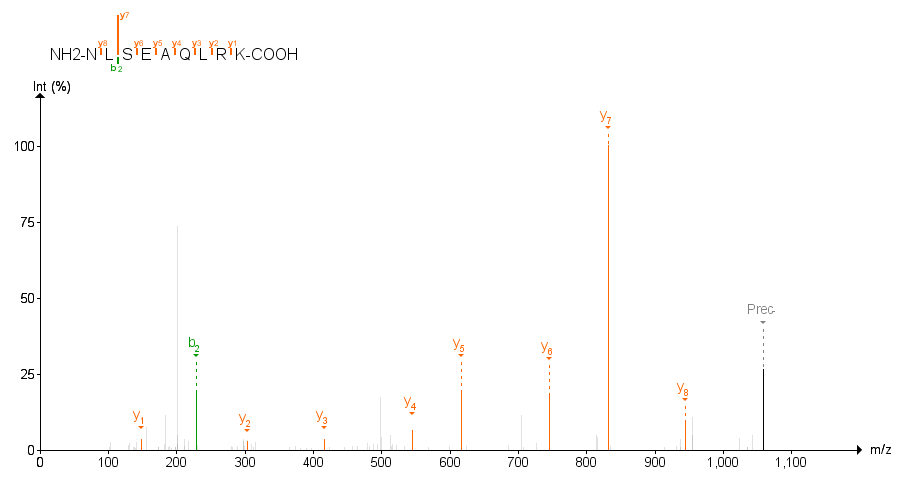


**Figure S17**. Tandem mass spectra for calmodulin-like protein 5 peptides annotated via MaxQuant version 1.5.4.0.


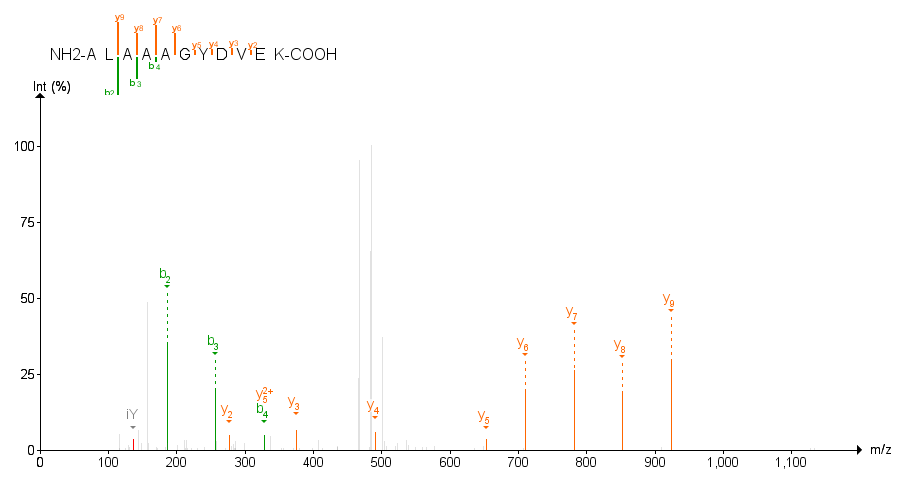


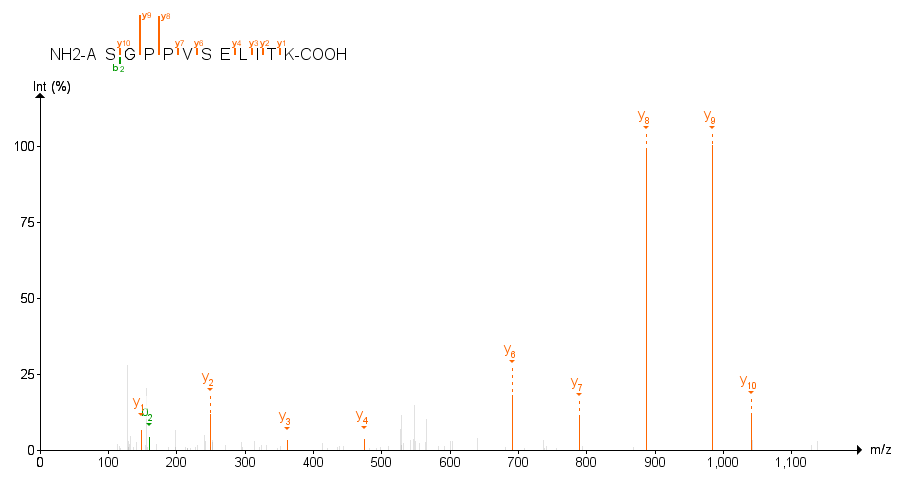


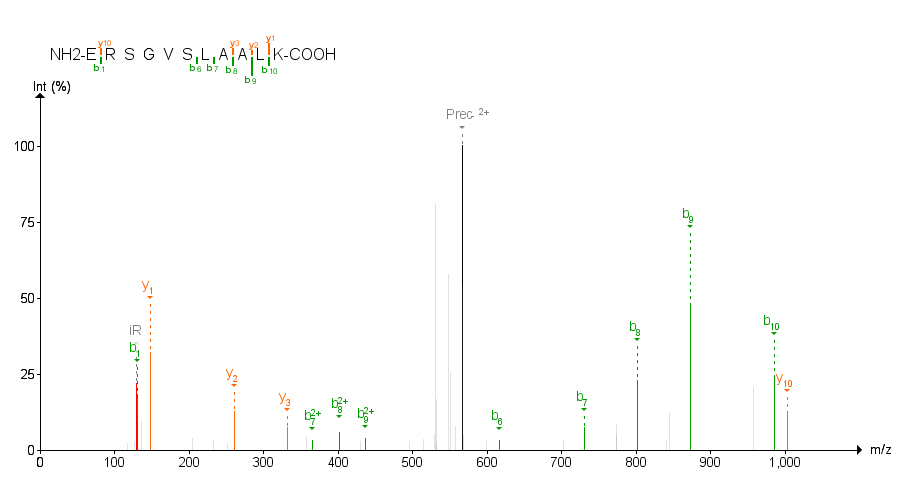


**Figure S18**. Tandem mass spectra for histone H1.2/H1.3/H1.4 peptides annotated via MaxQuant version 1.5.4.0.


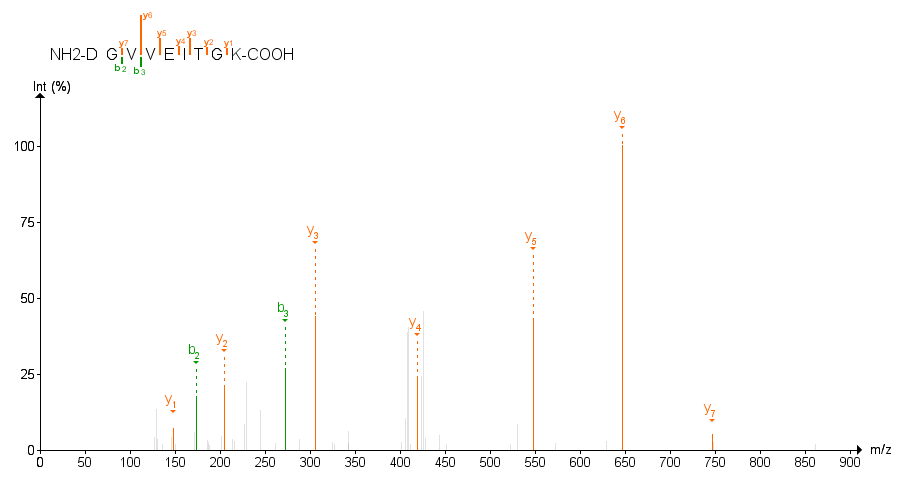


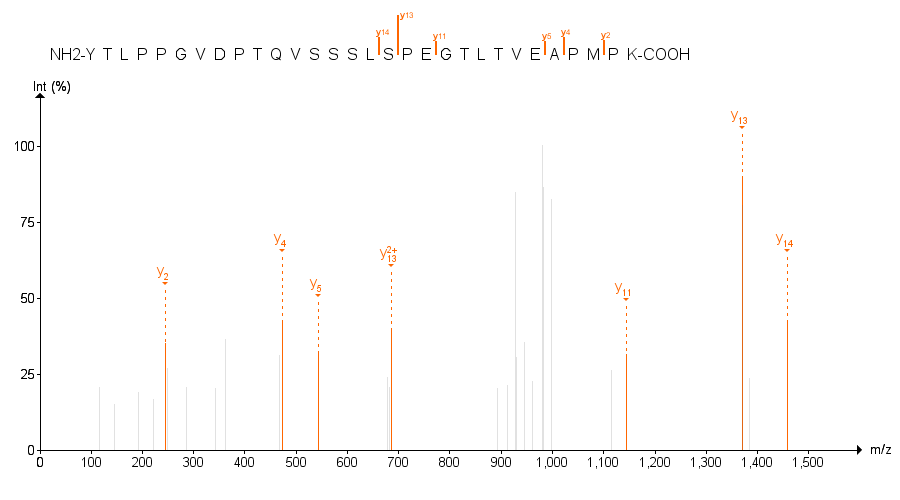


**Figure S19**. Tandem mass spectra for heat shock protein beta 1 peptides annotated via MaxQuant version 1.5.4.0.


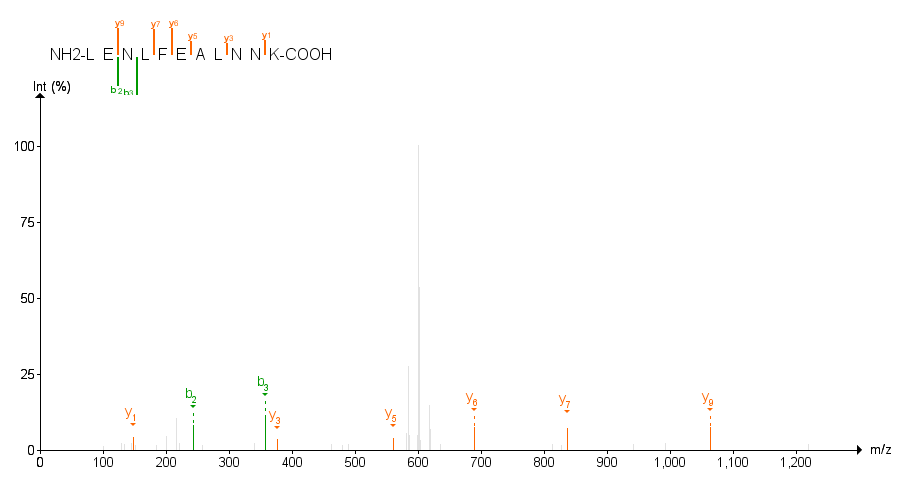


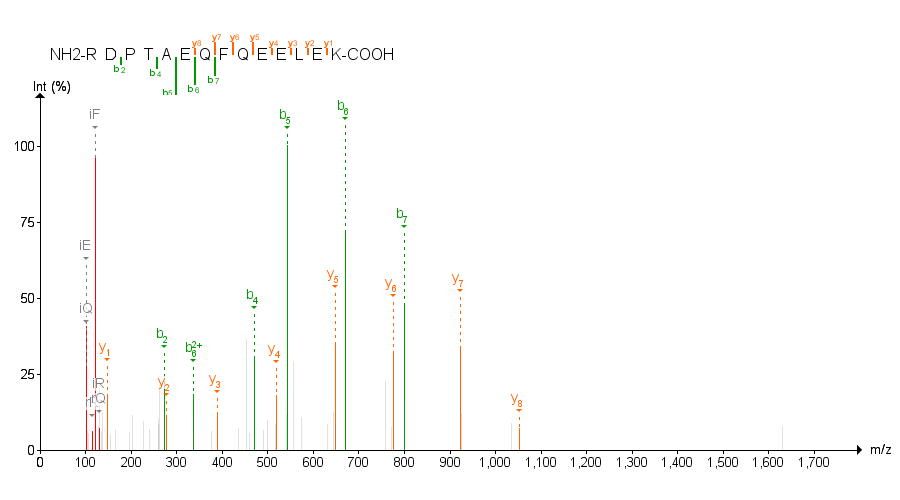


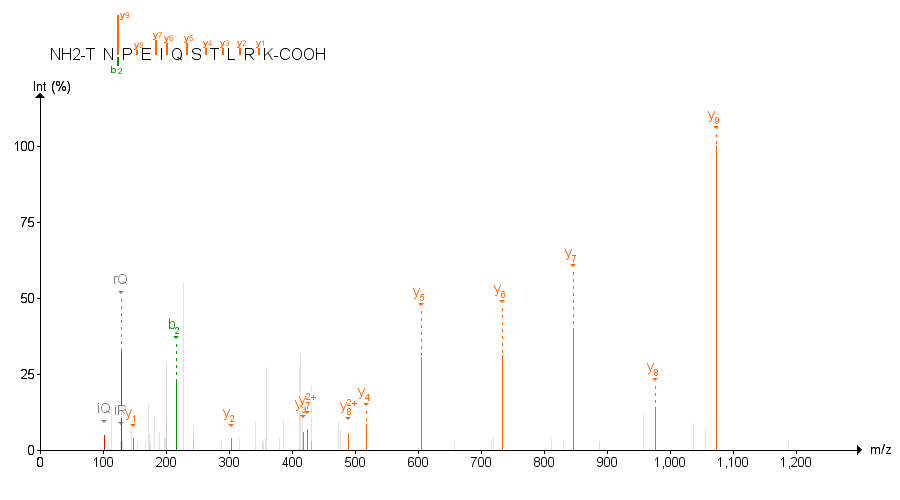


**Figure S20**. Tandem mass spectra for caspase-14 peptides annotated via MaxQuant version 1.5.4.0.


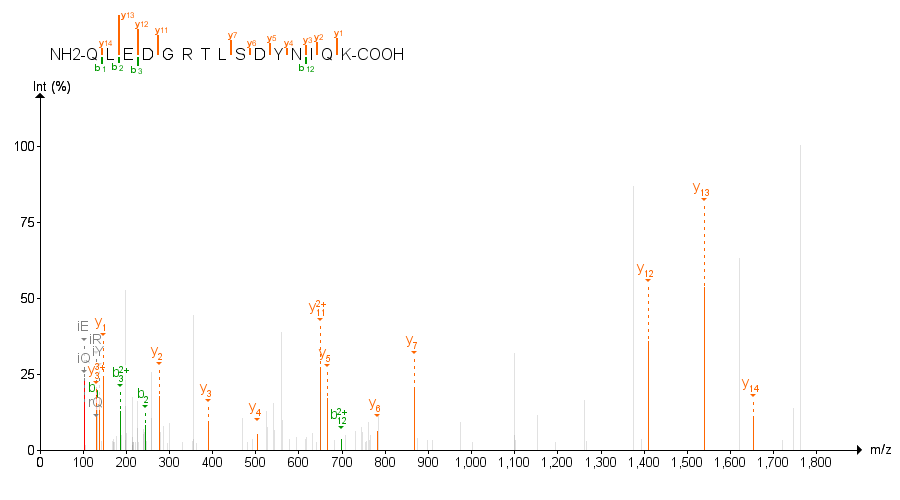


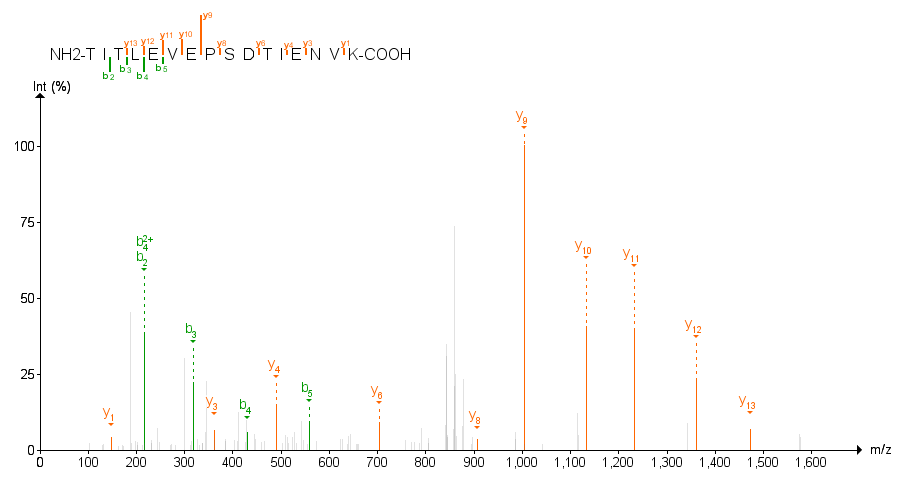


**Figure S21**. Tandem mass spectra for ubiquitin-60S ribosomal protein L40 peptides annotated via MaxQuant version 1.5.4.0.

| Mouse | Residual Standard Error | Multiple R^2^ | Adjusted R^2^ | p-value |
| --- | --- | --- | --- | --- |
| 901 | 2.599e-05 | 0.6288 | 0.6144 | 2.2e-16 |
| 902 | 2.881e-05 | 0.5584 | 0.5413 | 2.2e-16 |
| 903 | 2.017e-05 | 0.3573 | 0.3325 | 2.2e-16 |
| 904 | 2.437e-05 | 0.6930 | 0.6813 | 2.2e-16 |
| 905 | 3.160e-05 | 0.5812 | 0.5636 | 2.2e-16 |

**Table S1**: Multiple R^2^, adjusted R^2^, and p-value for each mouse based on linear models used for cystatin intensities in **Figure 2B**.

| Mouse | Residual Standard Error | Multiple R^2^ | Adjusted R^2^ | p-value |
| --- | --- | --- | --- | --- |
| 901 | 1.111e-05 | 0.6036 | 0.5873 | 2.2e-16 |
| 902 | 1.260e-05 | 0.2972 | 0.2690 | 2.812e-12 |
| 903 | 9.101e-06 | 4.125 | 0.3889 | 2.2e-16 |
| 904 | 1.329e-05 | 0.4048 | 0.3801 | 2.2e-16 |
| 905 | 1.443e-05 | 0.4207 | 0.3934 | 2.2e-16 |

**Table S2**: Multiple R^2^, adjusted R^2^, and p-value for each mouse based on linear models used for cystatin intensities in **Figure S1B**.

References

1. [Donato, R., Cannon, B. R., Sorci, G., Riuzzi, F., Hsu, K., Weber, D. J., et al. (2012) Functions of S100 Proteins. *Current Molecular Medicine* 13, 24–57](http://paperpile.com/b/NFdQ96/38Ktv)
2. [Wei, B.-R., Hoover, S. B., Ross, M. M., Zhou, W., Meani, F., Edwards, J. B., et al. (2009) Serum S100A6 concentration predicts peritoneal tumor burden in mice with epithelial ovarian cancer and is associated with advanced stage in patients. *PLoS One* 4, e7670](http://paperpile.com/b/NFdQ96/Zp8Jw)
3. [Bai, Y., Li, L.-D., Li, J., and Lu, X. (2018) Prognostic values of S100 family members in ovarian cancer patients. *BMC Cancer* 18, 1256](http://paperpile.com/b/NFdQ96/5N3kk)
4. [Xu, B., Chen, L., Zhan, Y., Marquez, K. N. S., Zhuo, L., Qi, S., et al. (2022) The Biological Functions and Regulatory Mechanisms of Fatty Acid Binding Protein 5 in Various Diseases. *Front Cell Dev Biol* 10, 857919](http://paperpile.com/b/NFdQ96/DcFEr)
5. [Gharpure, K. M., Pradeep, S., Sans, M., Rupaimoole, R., Ivan, C., Wu, S. Y., et al. (2018) FABP4 as a key determinant of metastatic potential of ovarian cancer. *Nat. Commun.* 9, 2923](http://paperpile.com/b/NFdQ96/dxUrn)
6. [Sun, N., and Zhao, X. (2022) Therapeutic Implications of FABP4 in Cancer: An Emerging Target to Tackle Cancer. *Front. Pharmacol.* 13, 948610](http://paperpile.com/b/NFdQ96/DWz9R)
7. [Liu, R.-Z., and Godbout, R. (2020) An Amplified Fatty Acid-Binding Protein Gene Cluster in Prostate Cancer: Emerging Roles in Lipid Metabolism and Metastasis. *Cancers*  12,](http://paperpile.com/b/NFdQ96/9qkJH)
8. [Lemberger, L., Wagner, R., Heller, G., Pils, D., and Grunt, T. W. (2022) Pharmacological Inhibition of Lipid Import and Transport Proteins in Ovarian Cancer. *Cancers*  14,](http://paperpile.com/b/NFdQ96/b17Jx)
9. [Krajewska, M., Kim, H., Shin, E., Kennedy, S., Duffy, M. J., Wong, Y. F., et al. (2005) Tumor-associated alterations in caspase-14 expression in epithelial malignancies. *Clin. Cancer Res.* 11, 5462–5471](http://paperpile.com/b/NFdQ96/EGyyr)
10. [Markiewicz, A., Sigorski, D., Markiewicz, M., Owczarczyk-Saczonek, A., and Placek, W. (2021) Caspase-14-From Biomolecular Basics to Clinical Approach. A Review of Available Data. *Int. J. Mol. Sci.* 22,](http://paperpile.com/b/NFdQ96/OnPMS)
11. [Joehlin-Price, A. S., Elkins, C. T., Stephens, J. A., Cohn, D. E., Knobloch, T. J., Weghorst, C. M., et al. (2014) Comprehensive evaluation of caspase-14 in vulvar neoplasia: an opportunity for treatment with black raspberry extract. *Gynecol. Oncol.* 135, 503–509](http://paperpile.com/b/NFdQ96/eKG5L)
12. [Villalobo, A., and Berchtold, M. W. (2020) The role of calmodulin in tumor cell migration, invasiveness, and metastasis. *Int. J. Mol. Sci.* 21, 765](http://paperpile.com/b/NFdQ96/D46dA)
13. [Kitazawa, S., Takaoka, Y., Ueda, Y., and Kitazawa, R. (2021) Identification of calmodulin-like protein 5 as tumor-suppressor gene silenced during early stage of carcinogenesis in squamous cell carcinoma of uterine cervix. *Int. J. Cancer* 149, 1358–1368](http://paperpile.com/b/NFdQ96/WhpLK)
14. [Gocher, A. M., Azabdaftari, G., Euscher, L. M., Dai, S., Karacosta, L. G., Franke, T. F., et al. (2017) Akt activation by Ca/calmodulin-dependent protein kinase kinase 2 (CaMKK2) in ovarian cancer cells. *J. Biol. Chem.* 292, 14188–14204](http://paperpile.com/b/NFdQ96/NsARt)
15. [Chen, Z., Sun, X., Xia, Z., Wang, J., Guo, N., and Zhang, Y. (2022) CaMKK2 Promotes the Progression of Ovarian Carcinoma through the PI3K/PDK1/Akt Axis. *Comput. Math. Methods Med.* 2022, 7187940](http://paperpile.com/b/NFdQ96/QRbVd)
16. [Ma, S., Yang, Y., Wang, C., Hui, N., Gu, L., Zhong, H., et al. (2009) Endogenous human CaMKII inhibitory protein suppresses tumor growth by inducing cell cycle arrest and apoptosis through down-regulation of the phosphatidylinositide 3-kinase/Akt/HDM2 pathway. *J. Biol. Chem.* 284, 24773–24782](http://paperpile.com/b/NFdQ96/Dq4d)
17. [Lee Motoyama, J.-P., Kim-Motoyama, H., Kim, P., Nakagama, H., Miyagawa, K., and Suzuki, K. (2007) Identification of dermcidin in human gestational tissue and characterization of its proteolytic activity. *Biochem. Biophys. Res. Commun.* 357, 828–833](http://paperpile.com/b/NFdQ96/Zx9cx)
18. [Smith, A. P., Hoek, K., and Becker, D. (2005) Whole-genome expression profiling of the melanoma progression pathway reveals marked molecular differences between nevi/melanoma in situ and advanced-stage melanomas. *Cancer Biol. Ther.* 4, 1018–1029](http://paperpile.com/b/NFdQ96/9RQdF)
19. [Stewart, G. D., Lowrie, A. G., Riddick, A. C. P., Fearon, K. C. H., Habib, F. K., and Ross, J. A. (2007) Dermcidin expression confers a survival advantage in prostate cancer cells subjected to oxidative stress or hypoxia. *Prostate* 67, 1308–1317](http://paperpile.com/b/NFdQ96/y5w63)
20. [Lowrie, A. G., Dickinson, P., Wheelhouse, N., Stewart, G. D., Ross, A. J., Forster, T., et al. (2011) Proteolysis-inducing factor core peptide mediates dermcidin-induced proliferation of hepatic cells through multiple signalling networks. *Int. J. Oncol.* 39, 709–718](http://paperpile.com/b/NFdQ96/PUUbV)
21. [Yarbrough, V. L., Winkle, S., and Herbst-Kralovetz, M. M. (2015) Antimicrobial peptides in the female reproductive tract: a critical component of the mucosal immune barrier with physiological and clinical implications. *Hum. Reprod. Update* 21, 353–377](http://paperpile.com/b/NFdQ96/5j9qe)
22. [Trzoss, L., Fukuda, T., Costa-Lotufo, L. V., Jimenez, P., La Clair, J. J., and Fenical, W. (2014) Seriniquinone, a selective anticancer agent, induces cell death by autophagocytosis, targeting the cancer-protective protein dermcidin. *Proc. Natl. Acad. Sci. U. S. A.* 111, 14687–14692](http://paperpile.com/b/NFdQ96/PLj8A)
23. [Júnior, L. A. L., Cucielo, M. S., Domeniconi, R. F., Dos Santos, L. D., Silveira, H. S., da Silva Nunes, I., et al. (2019) P-MAPA and IL-12 Differentially Regulate Proteins Associated with Ovarian Cancer Progression: A Proteomic Study. *ACS Omega* 4, 21761–21777](http://paperpile.com/b/NFdQ96/ROnJO)
24. [Ritossa, F. (1962) A new puffing pattern induced by temperature shock and DNP in drosophila. *Experientia* 18, 571–573](http://paperpile.com/b/NFdQ96/OC0aT)
25. [Matz, J. M., Blake, M. J., Tatelman, H. M., Lavoi, K. P., and Holbrook, N. J. (1995) Characterization and regulation of cold-induced heat shock protein expression in mouse brown adipose tissue. *Am. J. Physiol.* 269, R38–47](http://paperpile.com/b/NFdQ96/fJw4u)
26. [Cao, Y., Ohwatari, N., Matsumoto, T., Kosaka, M., Ohtsuru, A., and Yamashita, S. (1999) TGF-beta1 mediates 70-kDa heat shock protein induction due to ultraviolet irradiation in human skin fibroblasts. *Pflugers Arch.* 438, 239–244](http://paperpile.com/b/NFdQ96/0ZetU)
27. [Hoter, A., and Naim, H. Y. (2019) Heat Shock Proteins and Ovarian Cancer: Important Roles and Therapeutic Opportunities. *Cancers*  11,](http://paperpile.com/b/NFdQ96/MYgZF)
28. [Langdon, S. P., Rabiasz, G. J., Hirst, G. L., King, R. J., Hawkins, R. A., Smyth, J. F., et al. (1995) Expression of the heat shock protein HSP27 in human ovarian cancer. *Clin. Cancer Res.* 1, 1603–1609](http://paperpile.com/b/NFdQ96/cQLRK)
29. [Heiserman, J. P., Nallanthighal, S., Gifford, C. C., Graham, K., Samarakoon, R., Gao, C., et al. (2021) Heat Shock Protein 27, a Novel Downstream Target of Collagen Type XI alpha 1, Synergizes with Fatty Acid Oxidation to Confer Cisplatin Resistance in Ovarian Cancer Cells. *Cancers*  13,](http://paperpile.com/b/NFdQ96/EbitS)
30. [Geisler, J. P., Geisler, H. E., Tammela, J., Miller, G. A., Wiemann, M. C., and Zhou, Z. (1999) A study of heat shock protein 27 in endometrial carcinoma. *Gynecol. Oncol.* 72, 347–350](http://paperpile.com/b/NFdQ96/0mQvW)
31. [Geisler, J. P., Geisler, H. E., Tammela, J., Wiemann, M. C., Zhou, Z., Miller, G. A., et al. (1998) Heat shock protein 27: an independent prognostic indicator of survival in patients with epithelial ovarian carcinoma. *Gynecol. Oncol.* 69, 14–16](http://paperpile.com/b/NFdQ96/NMM92)
32. [Geisler, J. P., Tammela, J. E., Manahan, K. J., Geisler, H. E., Miller, G. A., Zhou, Z., et al. (2004) HSP27 in patients with ovarian carcinoma: still an independent prognostic indicator at 60 months follow-up. *Eur. J. Gynaecol. Oncol.* 25, 165–168](http://paperpile.com/b/NFdQ96/QD2VS)
33. [Zhao, M., Shen, F., Yin, Y. X., Yang, Y. Y., Xiang, D. J., and Chen, Q. (2012) Increased expression of heat shock protein 27 correlates with peritoneal metastasis in epithelial ovarian cancer. *Reprod. Sci.* 19, 748–753](http://paperpile.com/b/NFdQ96/QprLz)
34. [Schneider, J., Jimenez, E., Marenbach, K., Marx, D., and Meden, H. (1998) Co-expression of the MDR1 gene and HSP27 in human ovarian cancer. *Anticancer Res.* 18, 2967–2971](http://paperpile.com/b/NFdQ96/u5u7y)
35. [Tanaka, Y., Fujiwara, K., Tanaka, H., Maehata, K., and Kohno, I. (2004) Paclitaxel inhibits expression of heat shock protein 27 in ovarian and uterine cancer cells. *Int. J. Gynecol. Cancer* 14, 616–620](http://paperpile.com/b/NFdQ96/5yaLQ)
36. [Owen, S., Zhao, H., Dart, A., Wang, Y., Ruge, F., Gao, Y., et al. (2016) Heat shock protein 27 is a potential indicator for response to YangZheng XiaoJi and chemotherapy agents in cancer cells. *Int. J. Oncol.* 49, 1839–1847](http://paperpile.com/b/NFdQ96/MJQ3G)
37. [Zhao, M., Ding, J. X., Zeng, K., Zhao, J., Shen, F., Yin, Y. X., et al. (2014) Heat shock protein 27: a potential biomarker of peritoneal metastasis in epithelial ovarian cancer? *Tumour Biol.* 35, 1051–1056](http://paperpile.com/b/NFdQ96/VvZnG)
38. [Medrzycki, M., Zhang, Y., McDonald, J. F., and Fan, Y. (2012) Profiling of linker histone variants in ovarian cancer. *Front. Biosci.*  17, 396–406](http://paperpile.com/b/NFdQ96/EXo85)
39. [Marsh, D. J., Shah, J. S., and Cole, A. J. (2014) Histones and their modifications in ovarian cancer - drivers of disease and therapeutic targets. *Front. Oncol.* 4, 144](http://paperpile.com/b/NFdQ96/nA57l)
40. [Yang, Q., Yang, Y., Zhou, N., Tang, K., Lau, W. B., Lau, B., et al. (2018) Epigenetics in ovarian cancer: premise, properties, and perspectives. *Mol. Cancer* 17, 109](http://paperpile.com/b/NFdQ96/lI2Kv)
41. [Ashraf, A. H. M., Afroze, S., Osuji, G., Kayani, S., Colon, N., Pantho, A., et al. (2020) Epigenetic Modifications in Ovarian Cancer: A Review. *J. Cancer Treatment Diagn.* 4, 17–35](http://paperpile.com/b/NFdQ96/QTfw8)
42. [Matei, D., and Nephew, K. P. (2020) Epigenetic Attire in Ovarian Cancer: The Emperor’s New Clothes. *Cancer Res.* 80, 3775–3785](http://paperpile.com/b/NFdQ96/0ah9r)
43. [Hooda, J., Novak, M., Salomon, M. P., Matsuba, C., Ramos, R. I., MacDuffie, E., et al. (2019) Early Loss of Histone H2B Monoubiquitylation Alters Chromatin Accessibility and Activates Key Immune Pathways That Facilitate Progression of Ovarian Cancer. *Cancer Res.* 79, 760–772](http://paperpile.com/b/NFdQ96/zmFt9)
44. [Dickson, K.-A., Cole, A. J., Gill, A. J., Clarkson, A., Gard, G. B., Chou, A., et al. (2016) The RING finger domain E3 ubiquitin ligases BRCA1 and the RNF20/RNF40 complex in global loss of the chromatin mark histone H2B monoubiquitination (H2Bub1) in cell line models and primary high-grade serous ovarian cancer. *Hum. Mol. Genet.* 25, 5460–5471](http://paperpile.com/b/NFdQ96/kwCTt)
45. [Cai, Y., Jin, J., Swanson, S. K., Cole, M. D., Choi, S. H., Florens, L., et al. (2010) Subunit composition and substrate specificity of a MOF-containing histone acetyltransferase distinct from the male-specific lethal (MSL) complex. *J. Biol. Chem.* 285, 4268–4272](http://paperpile.com/b/NFdQ96/yc8i7)
46. [Smith, E. R., Pannuti, A., Gu, W., Steurnagel, A., Cook, R. G., Allis, C. D., et al. (2000) The drosophila MSL complex acetylates histone H4 at lysine 16, a chromatin modification linked to dosage compensation. *Mol. Cell. Biol.* 20, 312–318](http://paperpile.com/b/NFdQ96/KsnJz)
47. [Smith, E. R., Cayrou, C., Huang, R., Lane, W. S., Côté, J., and Lucchesi, J. C. (2005) A human protein complex homologous to the Drosophila MSL complex is responsible for the majority of histone H4 acetylation at lysine 16. *Mol. Cell. Biol.* 25, 9175–9188](http://paperpile.com/b/NFdQ96/DKSbQ)
48. [Liu, N., Zhang, R., Zhao, X., Su, J., Bian, X., Ni, J., et al. (2013) A potential diagnostic marker for ovarian cancer: Involvement of the histone acetyltransferase, human males absent on the first. *Oncol. Lett.* 6, 393–400](http://paperpile.com/b/NFdQ96/nwsKh)
49. [Cai, M., Hu, Z., Liu, J., Gao, J., Tan, M., Zhang, D., et al. (2015) Expression of hMOF in different ovarian tissues and its effects on ovarian cancer prognosis. *Oncol. Rep.* 33, 685–692](http://paperpile.com/b/NFdQ96/vYWjX)
